# Introducing AFS ([Al(SO_3_F)_3_]_*x*_) – a thermally stable, readily available, and catalytically active solid Lewis superacid†

**DOI:** 10.1039/d4sc01753f

**Published:** 2024-05-02

**Authors:** Johanna Schlögl, Ole Goldammer, Julia Bader, Franziska Emmerling, Sebastian Riedel

**Affiliations:** a Fachbereich Biologie, Chemie, Pharmazie, Institut für Chemie und Biochemie – Anorganische Chemie, Freie Universität Berlin Fabeckstraße 34/36 14195 Berlin Germany schjo@zedat.fu-berlin.de s.riedel@fu-berlin.de; b Department Materials Chemistry, Federal Institute for Material Research and Testing Richard-Willstätter-Straße 11 12489 Berlin Germany

## Abstract

Common Lewis superacids often suffer from low thermal stability or complicated synthetic protocols, requiring multi-step procedures and expensive starting materials. This prevents their large-scale application. Herein, the easy and comparably cheap synthesis of high-purity aluminium tris(fluorosulfate) ([Al(SO_3_F)_3_]_*x*_, AFS) is presented. All starting materials are commercially available and no work-up is required. The superacidity of this thermally stable, polymeric Lewis acid is demonstrated using both theoretical and experimental methods. Furthermore, its synthetic and catalytic applicability, *e.g.* in bond heterolysis reactions and C–F bond activations, is shown.

## Abstract 2

This paper introduces the thermally stable, solid Lewis superacid aluminium tris(fluorosulfate) (AFS), that is easy-to-synthesize from commercially available starting materials. Its applicability, *e.g.* in catalytic C–F bond activations, is shown.

## Graphical abstract

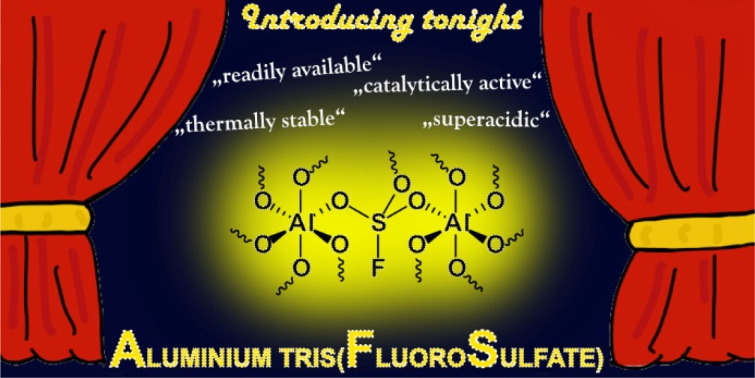

## Introduction

Trivalent aluminium species are archetypical Lewis acids and their applications in synthetic chemistry are manifold.^1,2^ Solid Lewis acids, like high-surface aluminium fluoride, HS-AlF_3_, or aluminium chlorofluoride (ACF, AlCl_*x*_F_3*x*_, with *x* = 0.3–0.05), serve as powerful catalysts in a variety of industrial transformations, *e.g.* C–H activation, C–F activation, (de)fluorination, or Friedel–Crafts type conversions.^2–4^ In fundamental research, the generation of highly reactive cations, *e.g.* [P_9_]^+^ ^5^ or [C(C_6_F_5_)_3_]^+^,^6^ has been realized using molecular Lewis acids like Al[OC(CF_3_)_3_]_3_ ^7,8^ or [Al(OTeF_5_)_3_]_2_.^9,10^ In the last two decades, the scope of aluminium-based Lewis acids has significantly broadened with the emergence of so-called Lewis superacids.^7–14^ These molecular Lewis acids outperform SbF_5_ in terms of acidity and handling. Furthermore, efforts to introduce superacidity to solid Lewis acids have been made, *e.g.* by treatment of partially dehydroxylated silica with Al[OC(CF_3_)_3_]_3_ ^15^ or by anion-doping of ACF using [Al(OTeF_5_)_3_]_2_.^16^ However, although the potential of these new Lewis superacids is well-acknowledged in fundamental research, their application in industry is all but popular. This is because they are either not easily accessible in bulk quantities, as they require multistep synthetic procedures involving rather expensive starting materials, or they exhibit low thermal stability.

In this context, the fluorosulfate group (–SO_3_F) presents an interesting ligand, as it can be introduced using comparably cheap, commercially available starting materials and it imposes great thermal stability on its compounds due to their tendency to polymerize. Compared to ACF, the bulkiness of the –SO_3_F group introduces a distortion to the three-dimensional network that could lead to enhanced Lewis acidity. Of the related trifluormethanesulfonate group (–SO_3_CF_3_, OTf) the Lewis acid Al(OTf_3_)_3_ is already known and well-established as a catalyst in a variety of organic transformations.^17^ However, quantum-chemical calculations render it to be non-superacidic.^11^

Aluminium tris(fluorosulfate) (Al(SO_3_F)_3_, AFS) was introduced already in 1983 by Verma and Singh.^18^ Preliminary reports on aluminium fluorosulfates included the partially substituted AlCl(SO_3_F)_2_ and the acetonitrile adduct Al(SO_3_F)_3_·3CH_3_CN, for none of which a characterization was provided.^19^ Verma and Singh published a synthetic route starting from amalgamated aluminium and HOC(O)CF_3_ and subsequent conversion of the obtained Al[OC(O)CF_3_]_3_ with 3 equivalents of HSO_3_F. However, all our attempts to reproduce this reaction have always resulted in an incomplete substitution (Fig. S1†). Only by introducing a large excess of HSO_3_F a full substitution could be achieved. However, removing the excess acid afterwards is a tedious task, which makes the whole procedure impractical, apart from its multi-step nature and the need for an amalgam (Fig. S2†). Thus, an alternative to Al[OC(O)CF_3_]_3_ as a starting material and a more practical route to the published synthesis needs to be developed.

Herein, we report on the preparation and isolation of AFS through an easy, straightforward process using AlMe_3_ and HSO_3_F, two commercially available and comparably cheap starting materials. Furthermore, the new synthetic protocol avoids the use of mercury for the first step, the activation of aluminium. The synthetic procedure requires no work-up and can be performed on a multigram scale. The polymeric nature and thermal stability of AFS is demonstrated and its superacidity is proven by applying both theoretical and experimental methods. Finally, its synthetic and catalytic applicability, *e.g.* in bond heterolysis reactions and C–F bond activations, is shown.

## Results and discussion

### Synthesis and characterization of AFS

**Scheme 1:**
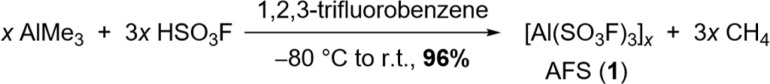
Synthesis of AFS (1).

The addition of 3 equivalents of HSO_3_F to a frozen solution of AlMe_3_ in 1,2,3-trifluorobenzene and subsequent warming of the mixture to room temperature leads to the evolution of methane and the formation of AFS (1) (Scheme 1). The latter can be isolated as a colourless powder in 96% yield after removal of all volatiles under reduced pressure and drying overnight.

The choice of solvent is crucial for the success of the synthesis of 1. While SO_2_Cl_2_ reacts with the starting materials, SO_2_ClF, a popular solvent in superacid chemistry, does not allow the warming of the reaction mixture to room temperature.^20^ Thus it results in an incomplete conversion yielding a temperature sensitive, in some cases explosive reaction product. Using *n*-pentane as a solvent leads to the formation of a bright yellow reaction mixture and the subsequent formation of a slurry, suggesting polymerization as a side reaction. Finally, standard aromatic solvents such as toluene or pyridine undergo electrophilic aromatic substitution (Fig. S4†). Hence, deactivated arenes need to be used as solvents. 1,2-Difluorobenzene seems to form adducts with the Lewis acid, as can be seen in the corresponding IR spectrum (Fig. S5†), so we opted for the even more strongly deactivated 1,2,3-trifluorobenzene.

1 is a colourless powder that can be stored for at least one year under an inert atmosphere at room temperature, as opposed to the reaction product obtained by Verma and Singh, which was only stable for a few days.^18^ TGA/DSC measurements reveal that the thermal decomposition of 1 occurs only at temperatures above 140 °C (Fig. S6†). 1 is sparingly soluble in acetonitrile and insoluble in HSO_3_F and most of the common inorganic and organic solvents (see ESI† for more information). This indicates a high degree of polymerization and therefore the formulation as [Al(SO_3_F)_3_]_*x*_ seems more appropriate than the formula based on composition. The polymeric structure is a common trait of metal fluorosulfates, due to the polydentate nature of the –SO_3_F ligand, and together with the high reactivity, this has often prevented their solid-state characterization by single-crystal X-ray diffraction.^21,22^ Only two molecular structures in the solid state are known of metal tris(fluorosulfates), Sb(SO_3_F)_3_ ^23^ and Au(SO_3_F)_3_.^24^ Indeed, powder XRD studies of 1 suggest an amorphous nature (Fig. S7†).

**Fig. 1:**
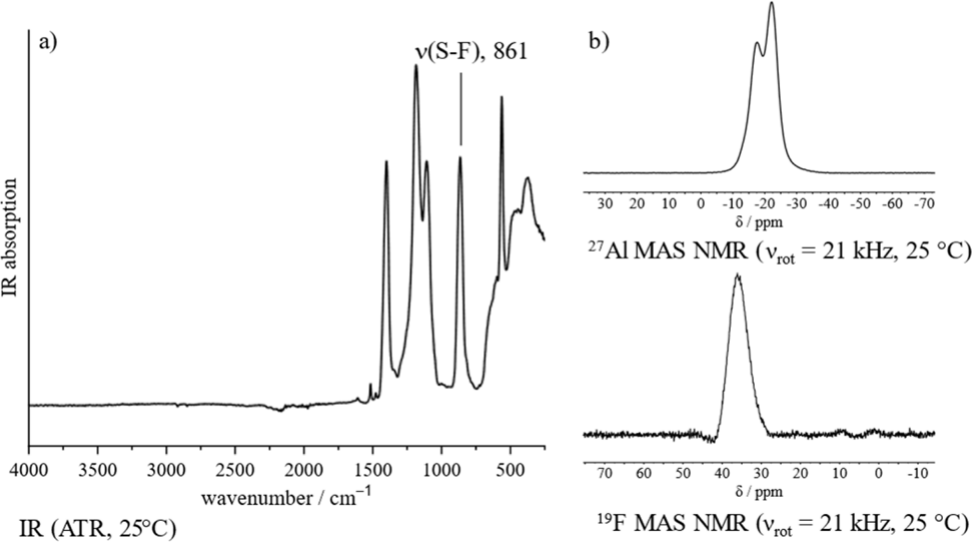
Spectroscopic characterization of 1: (a) IR spectrum (ATR, 25 °C). (b) ^27^Al and ^19^F MAS NMR spectra (*ν*_rot_ = 21 kHz, 25 °C).

The proposed polymeric structure of 1 is further supported by spectroscopic investigations. The IR spectrum shows a strongly blue-shifted S–F stretching band, indicating a covalent coordination of the –SO_3_F ligand as opposed to an ionic one (Fig. 1a).^21^ Though the spectrum contains six vibrational modes, suggesting a tridentate bridging –SO_3_F ligand,^21^ its band positions fit those of related polymeric Ga(SO_3_F)_3_ ^25^ and In(SO_3_F)_3_,^26^ which were assigned to be a bidentate bridging coordination mode. This is in agreement with the findings published by Verma and Singh.^18^ Taking into account our own investigations, a satisfactory assignment of the denticity of the –SO_3_F ligand is not possible, but a polymeric nature of 1 can be assumed.

The ^27^Al magic angle spinning (MAS) NMR spectrum of 1 shows two overlapping signals at −17 and −23 ppm that can be assigned to an octahedral coordination sphere around the aluminium (Fig. 1b).^27^ Due to the presence of strongly distorted [AlO_6_] moieties the signals are significantly broadened and indicate the presence of at least two different coordination polyhedra within the bulk. The ^19^F MAS NMR spectrum contains a broad singlet in the typical fluorosulfate region at 36 ppm (Fig. 1b).

**Fig. 2:**
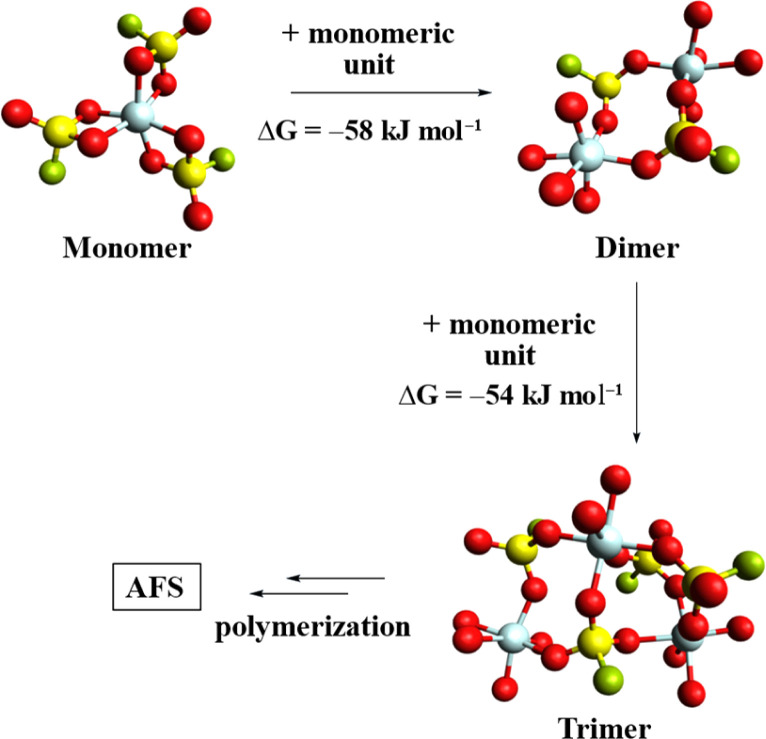
Calculated gas phase structures of [Al(SO_3_F)_3_]_*n*_ (*n* = 1, 2, 3). Some fluorosulfate ligands have been omitted for clarity and only their coordinated O-atoms are shown. (B3LYP-D3(BJ)/def2-TZVPP).

To further study the polymerization of 1, the gas-phase structures of the monomer, dimer, and trimer were calculated at the B3LYP-D3(BJ)/def2-TZVPP level (Fig. 2).^28–30^ A comparison of the respective Gibbs free energies reveals a free energy gain per monomer addition of roughly 56 kJ mol^−1^, suggesting that the polymerization of 1 is thermodynamically favoured.

### The theoretical Lewis acidity of Al(SO_3_F)_3_

To estimate the Lewis acidity of 1, monomeric Al(SO_3_F)_3_ was chosen as a simplified model and its gas-phase fluoride ion affinity (FIA) was computed at the BP86-D3(BJ)/def-SVP^28–30^ level using the isodesmic reaction with trimethylsilyl fluoride as anchor point.^31^

**Fig. 3:**
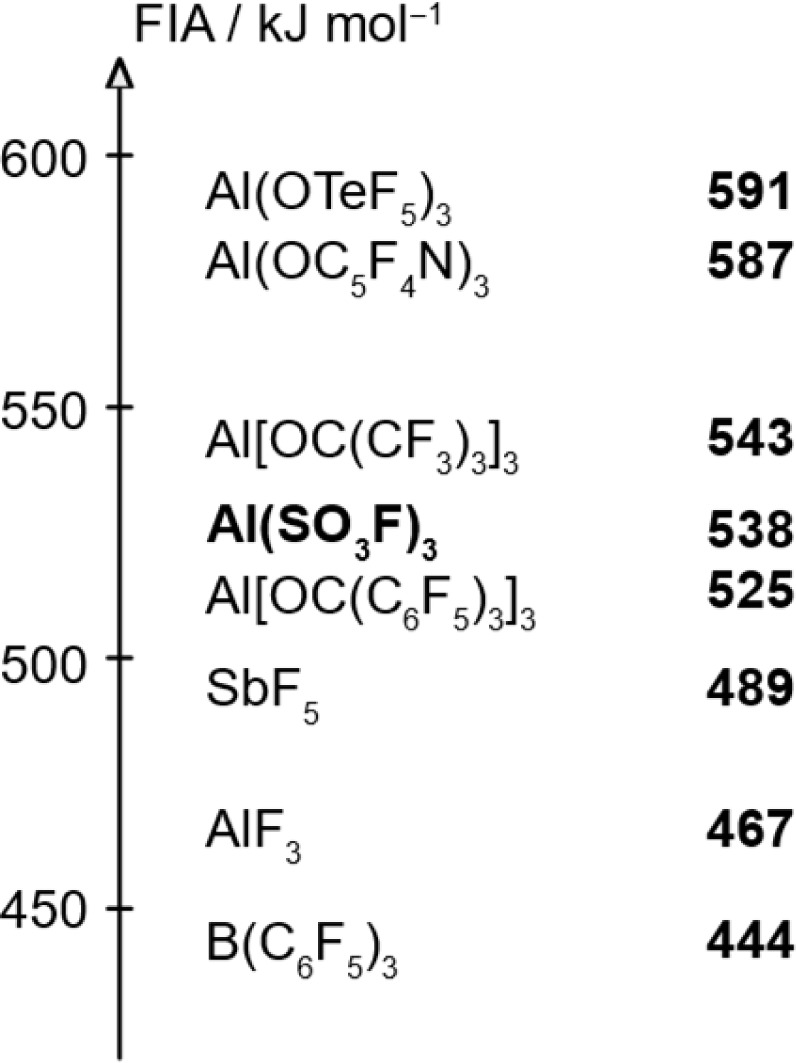
Calculated FIAs of selected Lewis acids.

The obtained FIA value of 538 kJ mol^−1^ is higher than the benchmark Lewis acid SbF_5_ with 489 kJ mol^−1^ and comparable to other aluminium-based Lewis superacids, thus theoretically rendering monomeric Al(SO_3_F)_3_ to be superacidic (Fig. 3).

### The experimental Lewis acidity of AFS

To evaluate the Lewis acidity of 1 in the condensed state, three different methods were applied: (I) investigating the blueshift of the C≡N stretching vibration of CD_3_CN upon adduct formation with AFS, (II) the Gutmann–Beckett method, and (III) a competition experiment with [PPh_4_][SbF_6_].

#### Investigation of the blue-shift of the C≡N stretching vibration of CD_3_CN upon adduct formation with AFS

The wavenumber of the C≡N stretching mode of CH_3_CN is a sensitive measure of Lewis acidity and its blueshift upon coordination to a Lewis acidic centre is frequently used for the evaluation of both solid and molecular Lewis acids.^11^ Fermi coupling between *ν*(CN) and *ν*(CC) + *δ*_s_(CH_3_) complicates the exact determination of Δ*ν*(CN) as it results in additional modes of medium intensity. Hence, the adduct with deuterated acetonitrile is normally prepared, as here no additional Fermi resonances appear. Upon coordination of CD_3_CN to 1, the C≡N stretching vibration of the adduct AFS·CD_3_CN (2) is blue-shifted by 78 cm^−1^ compared to free CD_3_CN (2336 cm^−1^, 2258 cm^−1^, Fig. S8†). This shift is higher than the one of SbF_5_·CD_3_CN (65 cm^−1^) and lies within the range of other aluminium-based Lewis superacids, such as Al[OC(C_6_F_5_)_3_]_3_ (79 cm^−1^) or [Al(OTeF_5_)_3_]_2_ (70 cm^−1^).^9,14,32^ Furthermore, it is higher than those of other aluminium-based solid Lewis acids, such as ACF (68 cm^−1^) or ACF teflate (73 cm^−1^).^16,33^

#### Gutmann–Beckett method

**Fig. 4:**
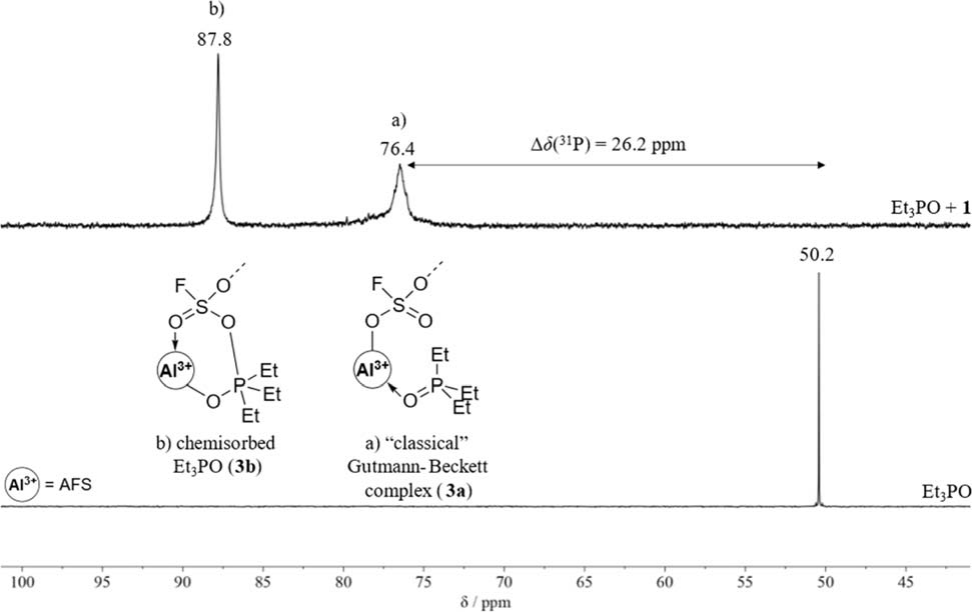
^31^P{^1^H} NMR spectrum of the reaction between Et_3_PO and 1 to determine the Lewis acidity of 1 by the Gutmann–Beckett method. Two species are initially formed: (a) the unstable “classical” Gutmann–Beckett complex (3a) and (b) the chemisorbed Et_3_PO unit within the polymer (3b). The ^31^P{^1^H} NMR spectrum of Et_3_PO is shown for comparison.

The change of the ^31^P NMR shift of Et_3_PO upon coordination to a Lewis acid is also known to correlate with Lewis acidity, which is known as the Gutmann–Beckett method.^34^ The triethylphosphine oxide adduct AFS·Et_3_PO (3) is prepared by adding one equivalent of Et_3_PO to a suspension of 1 in methylene chloride at room temperature. The ^31^P{^1^H} NMR spectrum of the reaction mixture reveals two signals: a broad resonance at 76.4 ppm, as well as a sharp one at 87.8 ppm (Fig. 4). We attribute the broad resonance at 76.4 ppm to Et_3_PO interacting with 1 as in the “classical” Gutmann–Beckett complex (3a). The chemical shift difference (Δ*δ*(^31^P)) of this resonance compared to uncoordinated Et_3_PO is 26.2 ppm, which is comparable to the values obtained for other aluminium-based Lewis superacids Al(C_6_F_5_)_3_: 26.0 ppm; Al[OC(C_6_F_5_)_3_]_3_: 23.9 ppm, ≡SiOAl[OC(CF_3_)_3_]_2_(O(Si≡)_2_: 28 ppm).^13–15^ The broadness of the resonance is likely due to some conformational flexibility within 3a leading to intramolecular rearrangements too fast to be discernible on the NMR timescale. Interestingly, after a few days, the resonance corresponding to 3a vanishes, whereas the sharp signal at 87.8 ppm remains. These observations may be explained by the freed-up coordination site of one –SO_3_F ligand upon coordination of the Et_3_PO moiety, inducing chemisorption of the Et_3_PO moiety. We therefore assign the signal at 87.8 ppm to chemisorbed Et_3_PO within the polymer (3b, Fig. 4). This is supported by the signal appearing at lower fields compared to 3a in the ^31^P{^1^H} NMR spectrum, which we attribute to the phosphorous atom being coordinated to two electron-withdrawing moieties within the polymer.

#### Competition experiment for fluoride ions with [PPh_4_][SbF_6_]

A direct experimental proof for Lewis superacidity can be obtained by performing a competition experiment with an [SbF_6_]^−^ salt, aiming for a fluoride abstraction from this anion. By combining 1 with [PPh_4_][SbF_6_] in acetonitrile at room temperature, a light-yellow solution is obtained. The corresponding ^19^F NMR spectrum reveals the formation of a fluoroaluminate along with [Sb_2_F_11_]^−^, the latter arising from the reaction between SbF_5_ and residual [SbF_6_]^−^ (Fig. S14†). This unambiguously proves a higher fluoride ion affinity of 1 compared to SbF_5_, and subsequently its superacidity.

### The reactivity of AFS

#### Application in bond-heterolysis reactions

**Scheme 2:**
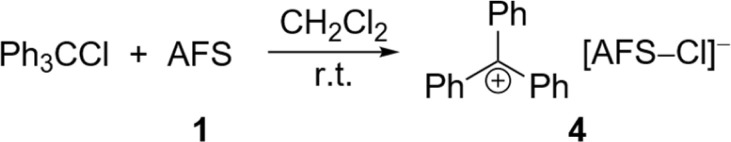
Synthesis of the trityl cation *via* chloride abstraction of Ph_3_CCl by 1.

The addition of trityl chloride, Ph_3_CCl, to a suspension of 1 in methylene chloride leads to the immediate formation of a luminous yellow solution suggesting the formation of a trityl cation, [CPh_3_]^+^ (Scheme 2), which was proven by ^1^H NMR spectroscopy (Fig. S16†). Despite the immediate colour change, the reaction took several days to complete, which can be attributed to the polymeric nature of 1 and its low tendency to solubilize, preventing a fast conversion. [Ph_3_C][AFS–Cl] (4) can be isolated as a yellow powder and stored indefinitely under inert conditions.

**Scheme 3:**
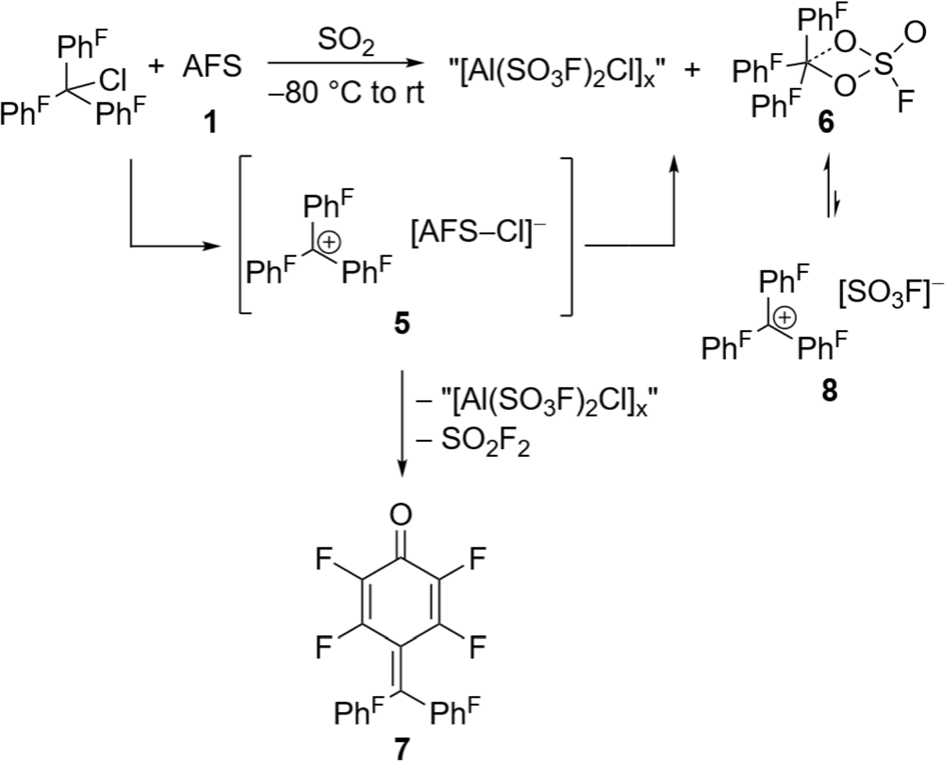
*In situ* generation of the [Ph^F^_3_C]^+^ cation *via* halide abstraction of perfluoro trityl chloride and further reactions leading to fluorosulfate 6 and ketone 7.

The perfluorinated analog of the trityl cation, [Ph^F^_3_C]^+^, was recently synthesized in our group through halide abstraction using the Lewis superacid [Al(OTeF_5_)_3_]_2_.^6^ This inspired us to test the effectiveness of 1 in a likewise reaction. Compared to its non-fluorinated analog, which is known as a versatile hydride and methanide abstraction agent, [Ph^F^_3_C]^+^ is expected to show an even higher reactivity.^35^ The addition of Ph^F^_3_CCl to a suspension of 1 in SO_2_ at −80 °C and subsequent warming of the reaction mixture to room temperature yields an intense red-violet suspension. The ^19^F NMR spectrum reveals four signals, that can be assigned to Ph^F^_3_COSO_2_F (6), with the fluorosulfate ligand bound to the central carbon (Fig. S19†). A similar outcome was observed by Dutton *et al.*, who isolated the respective Ph^F^_3_COSO_2_CF_3_ upon conversion of Ph^F^_3_CCl with stoichiometric amounts of HSO_3_CF_3_.^35^ The formation of 6 can be explained with the generation of the perfluorotrityl cation and [AFS–Cl]^−^ (5) and the subsequent decomposition to 6 (Scheme 3). In contrast to what we found in the case of 4, [AFS–Cl]^−^ is not stable in the presence of the strongly electrophilic [Ph^F^_3_C]^+^, which is likely why the abstraction of one –SO_3_F ligand occurs, followed by the attack at the central carbon atom. As a side reaction, the attack can also happen at the *para*-position of one perfluorinated aryl ring resulting in the formation of ketone 7 (Scheme 3, Fig. S20†). This reaction pathway can be followed by the appearance of five additional signals in the ^19^F NMR spectrum over time (Fig. S21†) and was also observed by Dutton *et al.*^35^ Intriguingly, the reaction mixture remains red-violet in color, though 6 should be colorless (Table S1†) and 7 is light-tan.^35^ Moreover, a similar red-violet color has been described multiple times with respect to the formation of [Ph^F^_3_C]^+^ and UV-Vis spectroscopy reveals an absorption maximum at *λ* = 500 nm, which is also the reported value for [Ph^F^_3_C]^+^ (Fig. S22†).^6,36^ Nevertheless, all efforts to detect signals corresponding to the cation *via* low-temperature ^19^F NMR spectroscopy failed. These observations infer that the –SO_3_F group in 6 is only loosely bound to the central carbon atom, owing to the highly delocalized charge and its polydentate nature, and that 6 is in an equilibrium with 8. However, this reaction is too fast to be discernible on the NMR timescale, yet detectable through UV-Vis spectroscopy.

In an effort to investigate the hydride abstraction ability of the reaction mixture, Ph_3_CH was added resulting in an immediate color change from red-violet to yellow. This color change is consistent with the formation of the trityl cation [Ph_3_C]^+^, which is further supported by ^1^H NMR (Fig. S23†).

#### Deoxygenation of triethylphosphine oxide

**Scheme 4:**
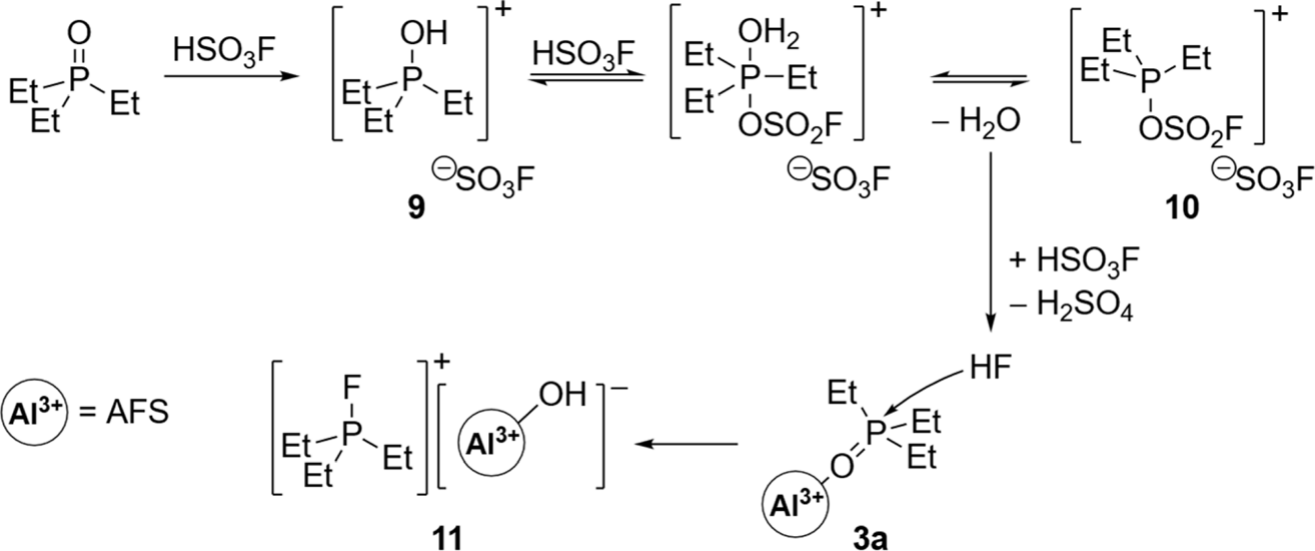
Proposed mechanism for the formation of fluorophosphonium salt 11. Residual HSO_3_F in the presence of Et_3_PO leads to the *in situ* formation of HF and subsequent attack of the Gutmann–Beckett complex 3a.

For the successful outcome of the aforementioned Gutmann–Beckett experiment, 1 must be completely free of any residual HSO_3_F. Otherwise, minute amounts of HSO_3_F will lead to the quantitative formation of the fluorophosphonium salt 11, as evidenced by the doublet resonance at 148.4 ppm (^1^*J*_PF_ = 970 Hz) in the corresponding ^31^P {^1^H} NMR spectrum (Fig. S26†).^37,38^ Close monitoring of the reaction mixture through ^31^P {^1^H} NMR spectroscopy over time indicates that the mechanism for the formation of 11 proceeds *via* phosphonium species 9 and 10, as well as the Gutmann–Beckett complex 3a (Scheme 4, S24†). The species 9 and 10 are generated *via* two subsequent proton transfer processes from HSO_3_F onto Et_3_PO. A similar process has been described by Pires and Fraile for Et_3_PO and HSO_3_CF_3_.^39^ In contrast to the latter, HSO_3_F is not water stable, thus the water molecule formed after the second proton transfer is consumed to afford HF and H_2_SO_4_. Intriguingly, in the absence of 1, the reaction between HSO_3_F and Et_3_PO stops at this point (Fig. S24†). However, in the presence of 1 deoxygenation of Et_3_PO occurs and the fluorophosphonium salt 11 is quantitatively formed (Fig. S24†). This can be explained by the activation of Et_3_PO in the presence of 1 through the formation of 3a, followed by the attack of the HF molecule. Only minute amounts of HSO_3_F are necessary in the beginning to start this reaction pathway, since more HSO_3_F is formed through the interaction of H_2_O and the water-instable AFS. A similar outcome of the Gutmann–Beckett experiment was previously reported for the Lewis acidic dications [(SIMes)PPh_2_F]^2+^ and [R(Ph_2_PF)_2_]^2+^ (R=C_10_H_6_, CH_2_), where an oxide-fluoride exchange yielded [PEt_3_F]^+^.^40^ Electrophilic phosphonium cations (ECPs) similar to [PEt_3_F]^+^ have been introduced by Stephan *et al.* as versatile main group catalysts, *e.g.* in the hydroarylation of olefins,^41^ isomerization and polymerizations of olefins or hydrosilylation of olefins or alkynes.^38,42^

#### Application of AFS as catalyst

**Fig. 5:**
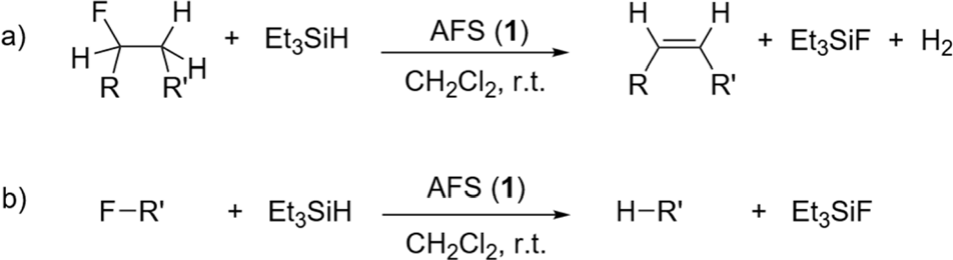
Catalytic C–F bond activation of fluoroalkanes in the presence of AFS and Et_3_SiH (R=H, alkyl; R′ = alkyl, aryl). (a) Dehydrofluorination (DHF), (b) hydrodefluorination (HDF).

The evidence of the Lewis superacidity and fluorophilicity of AFS prompted us to investigate its effectiveness as a Lewis acid catalyst. For that purpose, a qualitative evaluation of the catalytic activity of 1 was performed in stoichiometric dehydrofluorination (a) and hydrodefluorination (b) reactions using Et_3_SiH as the hydride donor (Fig. 5). A series of different fluoroalkanes, both primary and secondary, was tested and the reaction progress was followed *via*^19^F NMR spectroscopy (Table 1). Upon addition of Et_3_SiH to the reaction mixture containing 1 and the respective fluoroalkane at room temperature a vigorous reaction and the evolution of gaseous products was observed. We note that these are preliminary studies regarding the catalytic activity of 1 and that the nature and quantity of active sites have not been studied in detail and reaction conditions are not optimized.

**Table 1:** Catalytic C–F bond activation of fluoroalkanes at AFS^a^

Substrate	*t* [h]	Conv.^b^ [%]	C–F activation type	Main product^c^
1-Fluoropentane	2	>99	DHF	(*E*/*Z*)-2-Pentene
1-Fluoroheptane	2	61	DHF	*cis*/*trans*-2-Heptene
	24	>99		
2-Fluoropentane	1	>99	DHF	(*E*/*Z*)-2-Pentene
1-Fluoroadamantane^d^	1	>99	HDF	Adamantane
Fluorocyclohexane	1	>99	DHF/HDF	Cyclohexene, cyclohexane
2,2-Difluorobutane	2	59	DHF/HDF	(*E*)-2-Fluoro-2-butene, (*E*/*Z*)-2-butene
	24	80		
(CF_3_)C_6_H_5_	24	2	—	—

a 30 mg [46 mol% (100% active sites)] of the catalyst in a J Young NMR tube using CD_2_Cl_2_ as solvent (see ESI for more information).

b Conversions were determined through ^19^F NMR spectroscopy and are based on the converted fluorinated substrate into the corresponding products using CFCl_3_ as internal standard.

c In addition to Et_3_SiF.

d In this case only 10 mg [15 mol% (100% active sites)] of the catalyst were used.

At room temperature, 1-fluoropentane, 2-fluoropentane, and fluorocyclohexane were transformed quantitatively within 1–2 h through dehydrofluorination into the corresponding olefins, whereas 24 h were needed for 1-fluoroheptane. In the case of 1-fluoropentane and 1-fluoroheptane subsequent isomerization always yielded the respective 2-olefin. This high activity of AFS is remarkable, as for comparison, dehydrofluorination using ACF as catalyst and Et_3_GeH as hydride source only occurs at elevated temperatures.^4^ However, the conversion is not as fast as observed for ACF teflate catalysing the same reactions at room temperature.^16^ In the case of fluorocyclohexane, also hydrodefluorination occurred, leading to the formation of cyclohexane aside from cyclohexene. 1-Fluoroadamantane was consumed within one hour undergoing hydrodefluorination into adamantane. 2,2-Difluorobutane was mostly consumed within 24 h and was transformed almost selectively into (*E*)-2-fluoro-2-butene through dehydrofluorination. The latter partly underwent hydrodefluorination into (*E*/*Z*)-2-butene. Finally, in the case of trifluorotoluene almost no conversion could be observed, even after 24 h.

## Conclusion

In this work, we introduced a more convenient synthesis for high-purity AFS, which avoids the use of an aluminium amalgam. Our one-step synthesis relies on AlMe_3_ and HSO_3_F as commercially available and comparably cheap starting materials, and has the additional advantage that no work-up is required. The superacidity of this polymeric, thermally stable aluminium Lewis acid was demonstrated both computationally and experimentally and its applicability in typical Lewis acid transformations was shown. In bond heterolysis reactions it was not only possible to stabilize the trityl cation [Ph_3_C]^+^, but also to generate its perfluorinated analogue [Ph^F^_3_C]^+^. Moreover, AFS was able to deoxygenate Et_3_PO in the presence of HF to afford the corresponding fluorophosphonium cation [PEt_3_F]^+^. Finally, the high catalytic activity of AFS was successfully tested in dehydrofluorination and hydrodefluorination reactions at room temperature using Et_3_SiH as the hydride source. AFS arises as a new thermally stable, polymeric Lewis acid which is easy to manage, comparably cheap to prepare and which showcases superacidic character. With that, AFS builds a bridge between molecular Lewis superacids, which are either non-easily accessible or thermally unstable, and solid Lewis acids, which are normally not as acidic. In conclusion, we hope to pave the way for a Lewis superacid that is suitable for large-scale applications.

## Data availability

Additional details regarding experimental methods and experimental data are given in the ESI.†

## Author contributions

J. S. and O. G. performed synthetic work and collected vibrational spectroscopy data. J. S. and J. B. collected LT-NMR data. F. E. collected powder-XRD data. J. S. wrote the manuscript. All authors discussed and commented on the manuscript. J. S., J. B. and S. R. revised the manuscript. J. S. and S. R. conceptualized and coordinated the project.

## Conflicts of interest

There are no conflicts to declare.

## Supplementary Material

SC-015-D4SC01753F-s001
