# Supplementary material for: Introducing AFS ([Al(SO3F)3]x) – a thermally stable, readily available, and catalytically active solid Lewis superacid
Source: Chem Sci. 2024 May 2;15(21):8038–44. doi: 10.1039/d4sc01753f (PMC11134397; doi:10.1039/d4sc01753f)
Supplement: SC-015-D4SC01753F-s001 [file SC-015-D4SC01753F-s001.pdf]

Electronic Supplementary Material (ESI) for Chemical Science.
This journal is © The Royal Society of Chemistry 2024

Supporting Information

1. General Information 2
2. Syntheses and spectral data 3
   2.1. Literature procedure.[6] 3
   2.2. Preparation of AFS (1) 4
3. Testing the Lewis acidity of AFS 8
   3.1. AFS·CD_3CN (2) 8
   3.2. Gutmann-Beckett method. 10
4. Testing the reactivity of AFS 14
   4.1. Halide Abstraction Reaction with Ph_3CCl. 14
   4.2. Halide Abstraction Reaction with $Ph^F{}_3CCl$. 16
   4.3. Deoxygenation of Et_3PO 19
5. Testing the catalytic activity 22
   5.1. General procedure for C−F activation reactions 22
   5.2. NMR spectra of C−F activation reactions 22
6. Quantum-chemical calculations 30
7. Literature 34

1. General Information

All preparative work was carried out under an inert atmosphere using standard Schlenk techniques. Glassware was greased with Triboflon III. HSO_3F was transferred using self-made PFA cannulas. All solid materials were stored and handled inside a glove box with an atmosphere of dry argon (O_2 < 0.5 ppm, H_2O < 0.5 ppm). All solvents were dried on CaH_2 (except for acetonitrile which was dried over P_4O_{10}) and stored on molecular sieve. HSO_3F was distilled before use and kept in a PFA bottle at 4 °C. Trimethylaluminum in hexane (2M) was purchased from Sigma Aldrich and used as provided. Trifluoroacetic acid (TFA) and TFA anhydride were purchased from abcr and used as provided. CPh_3Cl was sublimed and dried in high vacuum. $C(C_6F_5)_3Cl$ was prepared according to a literature procedure.[1] IR spectra were measured on a Bruker ALPHA FTIR spectrometer equipped with a diamond ATR attachment in a glove box filled with argon (resolution 4 cm^{-1}). Liquid NMR spectra were recorded on a JEOL 400 MHz ECS or ECZ spectrometer in J. Young NMR tubes. All reported chemical shifts were referenced to the Ξ values given in IUPAC recommendations of 2008 using the ^2H signal of the deuterated solvent as internal standard. External c Solid-state MAS (magic-angle spinning) nuclear magnetic resonance spectra were recorded on a JEOL 600 MHz ECZ S spectrometer. Thermogravimetric analyses (TGA) and differential scanning calorimetry (DSC) measurements were conducted using a NETZSCH STA 449 F3 Jupiter®. The sample was heated with a temperature gradient of 10 K min^{-1}. UV-vis spectra were recorded using a Perkin Elmer Lambda 465 photometer with deuterium and tungsten lamps. A quartz cuvette with a Rydberg-Schlenk attachment was used to maintain an inert set-up.

The *Turbomole*[2] program was used to perform structural optimizations at the unrestricted Kohn-Sham DFT level, using the BP86 or B3LYP hybrid functional (with RI) in conjunction with basis sets def-SV(P) and def2-TZVPP.[3] Minima on potential energy surfaces were characterized by normal mode analysis. Thermochemical data is provided without counterpoise correction but includes zero-point energy correction as obtained from harmonic vibrational frequencies. All stationary points of the potential energy surface (PES) were characterized by analytic evaluation of second derivatives. Optimized structures correspond to minima on their respective potential energy surfaces. Subsequent single point calculations were performed with the *ORCA*[4] program package. For FIA calculations all compounds were computed in the gas phase, for oligomerization reactions and TD-DFT calculations the COSMO and the CPCM solvent model were used.[5]

2. Syntheses and spectral data

2.1. Literature procedure.[6]

Synthesis of $Al(O_2CCF_3)_3$. Freshly prepared aluminum amalgam (Al foil, 2% aqueous $HgCl_2$) was dissolved in aqueous TFA (50%). The clear solution was separated from liquid Hg and evaporated to dryness. A glassy substance was obtained, that was washed (2x) with TFA anhydride and dried in high vacuum. $Al(O_2CCF_3)_3$ was obtained as a colorless powder (2.2 g from 0.22 g Al).

IR (ATR, 25°C) $\tilde{v}$ = 3670 (w), 1672 (s, br), 1609 (vw, sh), 1507 (m), 1208 (vs), 1162 (vs), 1048 (s), 883 (w), 871 (w), 801 (m), 735 (m), 701 (w), 640 (m, br), 560 (s, br), 526 (m, sh), 453 (m), 432 (s) cm^{-1}.

Synthesis of "$Al(SO_3F)_3$". Freshly distilled HSO_3F (0.165 g, 1.65 mmol, 3.0 eq) was added to a solution of $Al(O_2CCF_3)_3$ (0.2 g, 0.55 mmol) in SO_2Cl_2 (5 mL) at −140 °C. The reaction mixture was slowly warmed to 10 °C and stirred for 6 hours. It was filtrated and washed (2x) with SO_2Cl_2 and CCl_4 (1x) and dried overnight at high vacuum. A colorless powder was obtained.

In the "optimized" version of this procedure, 7 eq. HSO_3F were added to a solution of $Al(O_2CCF_3)_3$ in SO_2Cl_2.

IR (ATR, 25°C) $\tilde{v}$ = 3673 (w), 2947 (vb, w), 2513 (b, sh), 1684 (m), 1509 (w), 1403 (sh), 1330 (sh), 1213 (s), 1167 (vs), 1094 (vs), 1053 (sh), 990 (sh), 954 (m), 856 (m), 801 (m), 735 (m), 645 (sh), 560 (vs) cm^{-1}.

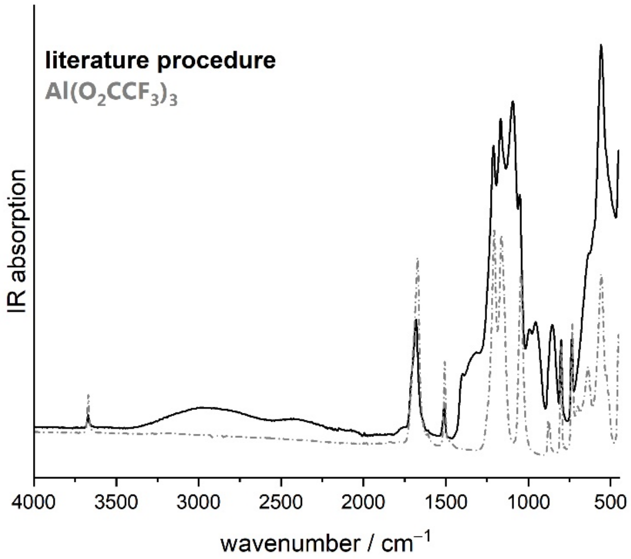

Figure S 1. IR (ATR, 25 °C) of the reaction product of the literature procedure. In grey, the IR spectrum of the starting material, $Al(O_2CCF_3)_3$, is shown. The reaction product does still contain starting material; indicating an incomplete conversion.

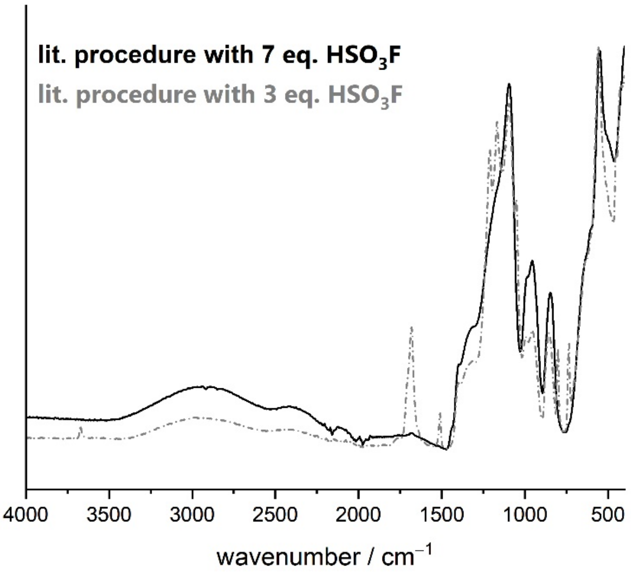

Figure S 2. IR (ATR, 25 °C) of the reaction product of the “optimized” literature procedure using a large excess of HSO_3F. No bands corresponding to the starting material $Al(O_2CCF_3)_3$ are visible anymore, yet the bands corresponding to fluorosulfuric acid are still present even after a long drying period.

2.2. Preparation of AFS (1)

A cooled solution of fluorosulfuric acid (450 mg, 4.5 mmol, 3.0 eq) in 1,2,3-trifluorobenzene was transferred onto a frozen solution of $AlMe_3$ (2M, in hexane, 108 mg, 1.5 mmol) in 1,2,3-trifluorobenzene at −80 °C. The mixture was first warmed to −30 °C and vigorously stirred until the gas evolution ceased. Afterward, the reaction mixture was brought to room temperature. A colorless solid precipitated. The solution was stirred for an additional hour at room temperature. Then, all volatiles were pumped off and the obtained colorless powder was dried overnight under high vacuum at 40 °C (465 mg, 1.44 mmol, 96%).

IR (ATR, 25 °C): ṽ = 1399 (s), 1380 (vs), 1105 (s), 862 (s), 562 (vs), 460 (m), 363 (s) cm^{-1}. **^{27}Al MAS NMR (21 kHz, 25 °C):** δ = −17 (s), −23 (s) ppm. **^{19}F MAS NMR (21 kHz, 25 °C):** δ = 36 (s) ppm.

**solvent**	solubility
MeCN, EtCN	sparingly soluble, but formation of various complexes, such as $[Al(SO_3F)_4(MeCN)_2]^-$ and $[Al(SO_3F)_2(MeCN)_4]^+$ due to autoionization as well as higher charged species
Et_2O, DCM, fluorobenzene, trifluorobenzene	no solubility
toluene, pyridine	soluble, but aromatic substrates undergo electrophilic aromatic substitution

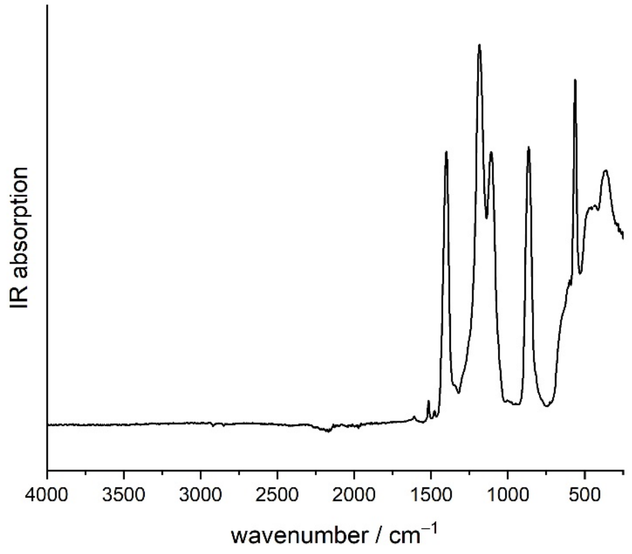

Figure S 3. IR (ATR, 25 °C) of **1**. The absence of bands around 3000 cm^{-1} and 960 cm^{-1} indicates the absence of residual HSO_3F.

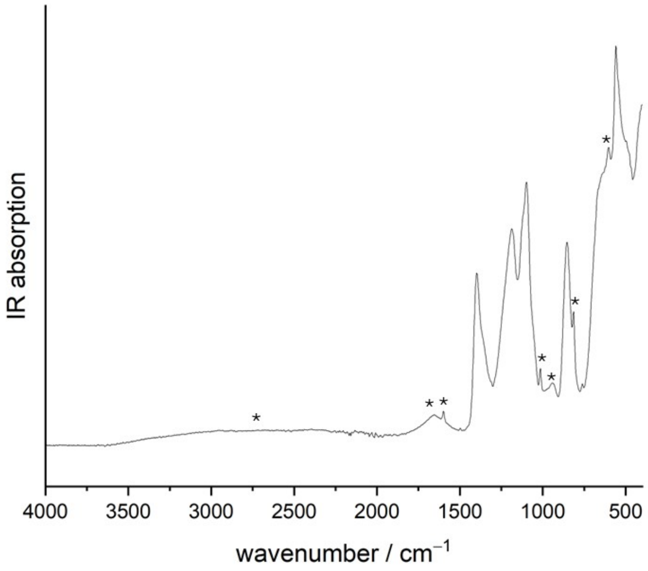

Figure S 4. IR (ATR, 25 °C) of **1** using toluene as a solvent for the synthesis. Bands corresponding to residual HSO_3F, as well as *p*-toluenesulfonic acid (which was isolated as colorless crystals from the reaction mixture) are marked with an asterisk. This indicates that electrophilic aromatic substitution is happening as a side reaction.

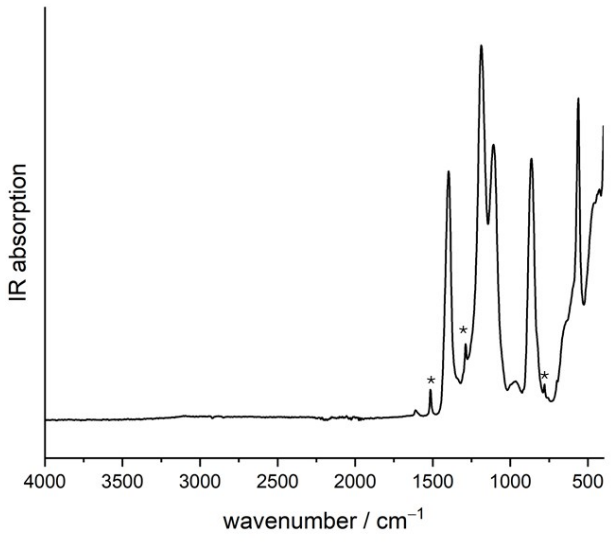

Figure S 5. IR (ATR, 25 °C) of **1** using oDFB as a solvent for the synthesis. Residual bands (marked with asterisks) of the solvent are visible still after hours of drying in high vacuum suggesting the formation of a solvent adduct. Compared to neat oDFB the bands are shifted by about 20-30 cm^{-1}.

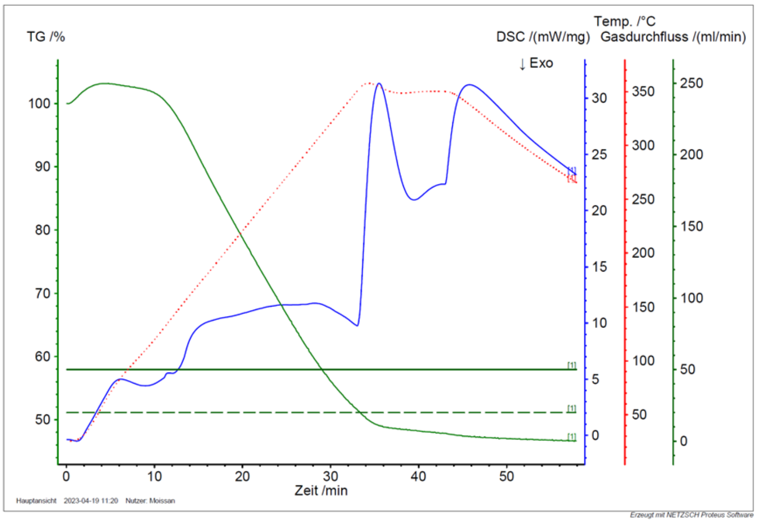

Figure S 6. Thermogravimetric analysis (green curve) and differential scanning calorimetry (blue curve) of **1**.

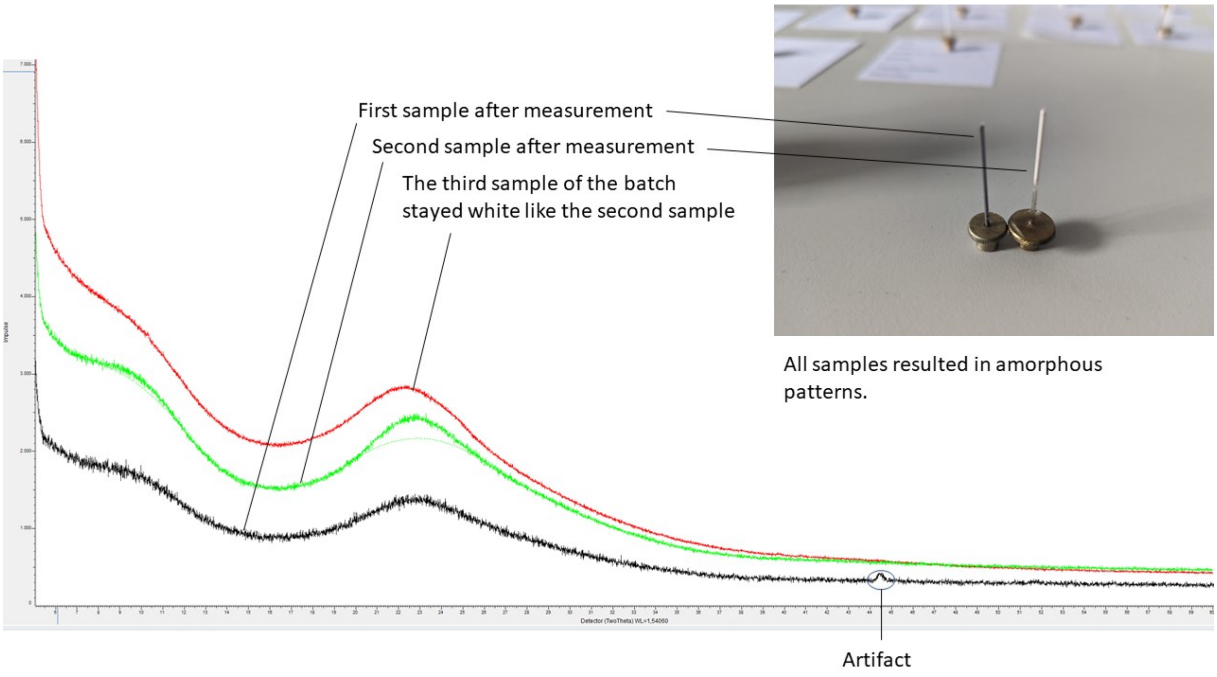

Figure S 7.: Powder XRD of **1**.

3. Testing the Lewis acidity of AFS

3.1. AFS·CD_3CN (2)

Fluorosulfuric acid (450 mg, 4.5 mmol, 3 eq.) was transferred onto a frozen solution of $AlMe_3$ (2M, in hexane 108 mg, 1.5 mmol) in deuterated acetonitrile at −80 °C. The mixture was gradually warmed to room temperature and a colorless solution was obtained. The solution was vigorously stirred for an additional hour at room temperature until all gas evolution had ceased. Afterwards, all volatiles were pumped off leading to the isolation of a colorless solid (520 mg).

IR (ATR, 25 °C): ṽ = 3010 (vw), 2943 (vw), 2336 (w), 2309 (vw), 1372 (m), 1303 (m), 1229 (s), 1066 (vs), 940 (w), 785 (s), 665 (vs), 558 (vs) cm^{-1}. **^1H NMR (400 MHz, acetonitrile-d$_3$, 25 °C):** δ = 2.0 (s, MeCN) ppm. **^{19}F NMR (377 MHz, acetonitrile-d$_3$, 25 °C):** δ = 39.6 (s, $[Al(SO_3F)_4(MeCN)_2]^-$), 38.9 (s, $[Al(SO_3F)_3(MeCN)_3]$), 38.6 (s, $[Al(SO_3F)_2(MeCN)_4]^+$) ppm. **^{27}Al NMR (104 MHz, acetonitrile-d$_3$, 25 °C):** δ = −15 (s), −22 (m) ppm.

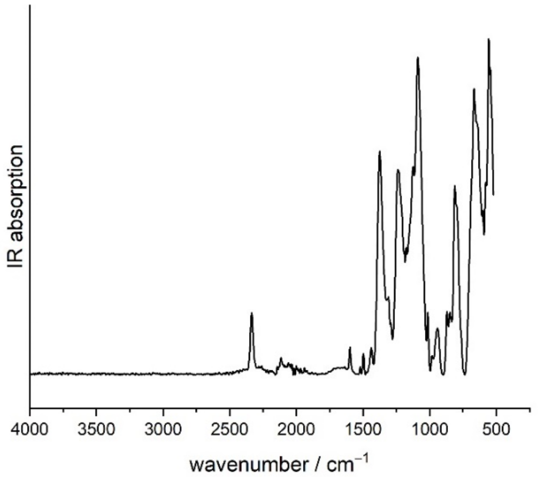

Figure S 8. IR (ATR, 25 °C) of **2**. ṽ (C≡N) = 2336 cm^{-1}.

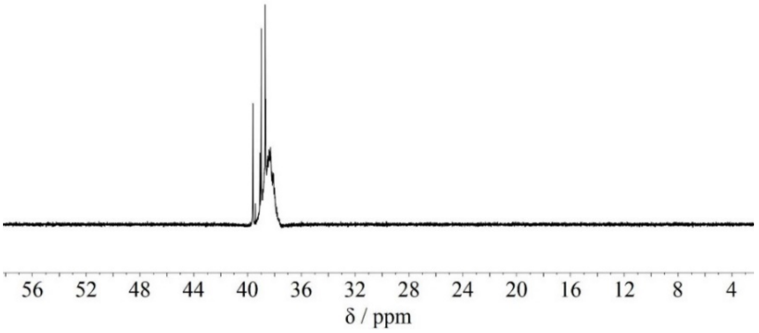

Figure S 9. ^{19}F NMR (377 MHz, acetonitrile-d$_3$, 25 °C) of **2**. The autoionization of **2** is visible, a phenomenon previously described for other nitrile adducts of Lewis acids.[7]

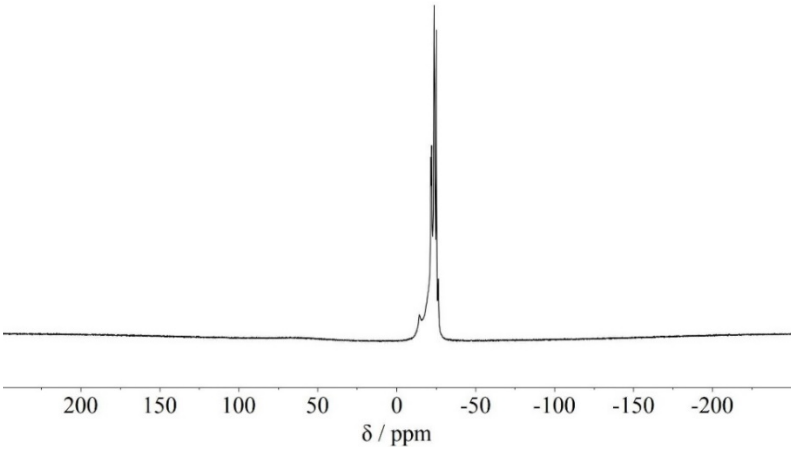

Figure S 10. ^{27}Al NMR (104 MHz, acetonitrile-d_3, 25 °C) of **2**. The autoionization of **2** is visible, a phenomenon previously described for other nitrile adducts of Lewis acids.[7]

3.2. Gutmann-Beckett method.

A solution of $OPEt_3$ (3 mg, 0.22 mmol, 1 eq.) in CH_2Cl_2 was added to **1** (7 mg, 0.22 mmol) at room temperature. A clear solution was obtained, that was stirred for 10 minutes. All volatiles were removed in vacuo leading to the isolation of a colorless powder.

^{1}H NMR (400 MHz, methylene chloride-d$_2$, 25 °C): δ = 2.13 (m, 6H, -$OPEt_3$), 1.31 (m, 9H, -$OPEt_3$) ppm. **^{19}F NMR (377 MHz, methylene chloride-d$_2$, 25 °C):** δ = 37.2 (s, br) ppm. **^{27} Al NMR (107 MHz, methylene chloride-d$_2$, 25 °C):** δ = −14 (s) ppm. **^{31}P {^{1}H} NMR (162 MHz, methylene chloride-d$_2$, 25 °C):** δ = 87.8 (s), 76.4 (s, br) ppm.

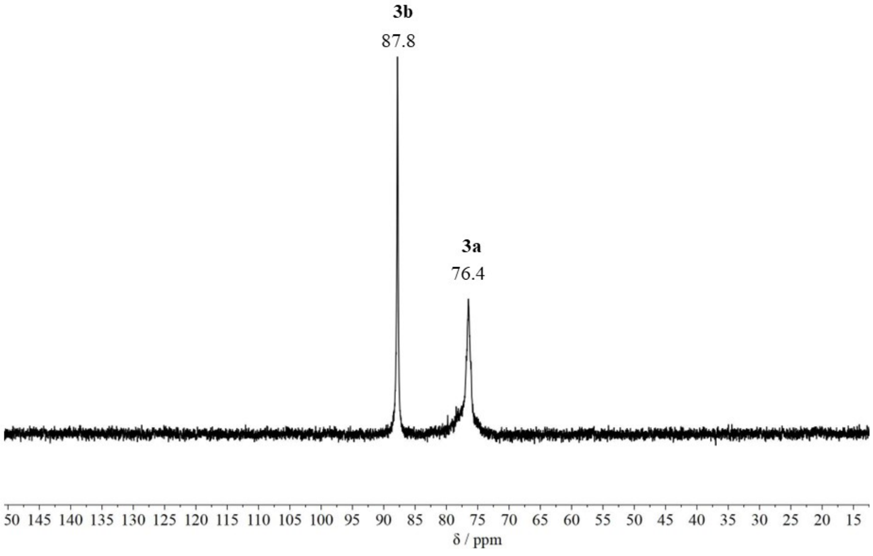

Figure S 11. ^{31}P{^{1}H} NMR (162 MHz, methylene chloride-d$_2$, 25 °C) of **3**. The spectrum shows one signal at higher fields corresponding to Et_3PO interacting with **1** (**3a**) and one signal at lower fields corresponding to chemisorbed Et_3PO (**3b**).

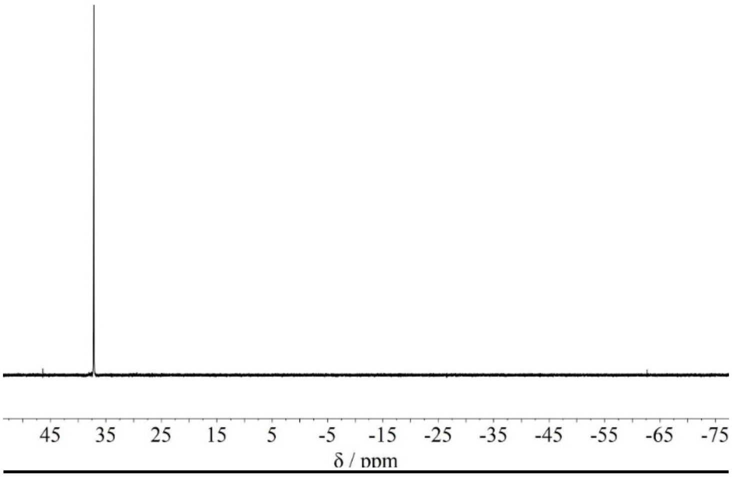

Figure S 12. ^{19}F NMR (377 MHz, methylene chloride-d$_2$, 25 °C) of **3**.

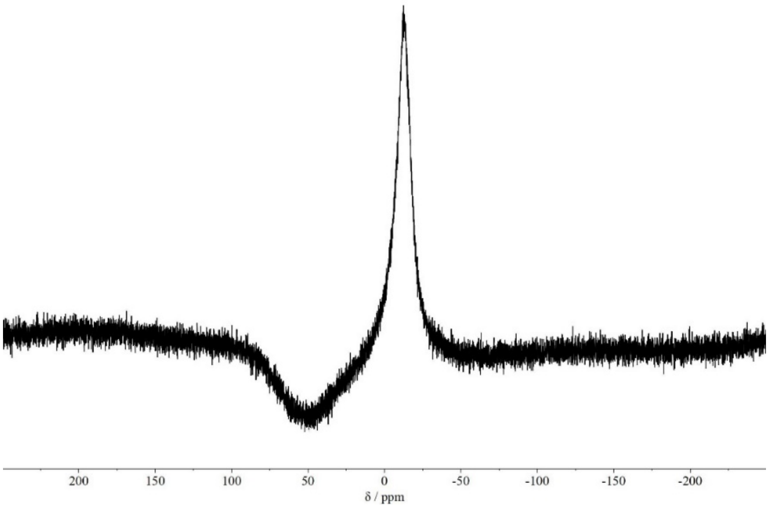

Figure S 13.: ^{27}Al NMR (107 MHz, methylene chloride-d_2, 25 °C) of **3**.

3.3. Competition experiment with $[PPh_4][SbF_6]$.

Stochiometric amounts of **1** (10 mg, 0.030 mmol) and $[PPh_4][SbF_6]$ (17 mg, 0.30 mmol) were placed in a *J. Young* NMR tube and mixed in acetonitrile. A yellowish solution was obtained.

^{1}H NMR (400 MHz, acetonitrile-d$_3$, 25 °C): δ = 7.95 (m, 4H, para-H, $[PPh_4]^+$), 7.78 (m, 8H, meta-H, $[PPh_4]^+$), 7.73 (m, 8H, ortho-H, $[PPh_4]^+$) ppm. **^{19}F NMR (377 MHz, acetonitrile-d$_3$, 25 °C):** δ = 39.3 (s), 38.6 (s), 37.0 (s, br), −114.2 (s), −126.8 (m), −132.1 (m), −158.9 (m) ppm. **^{27}Al NMR (107 MHz, acetonitrile-d$_3$, 25 °C):** δ = −15 (s) ppm.

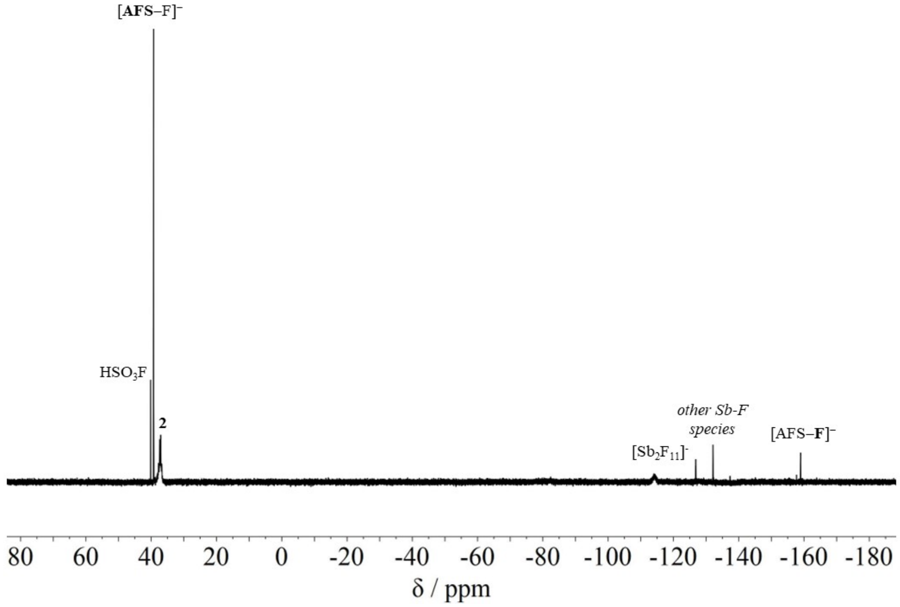

Figure S 14. ^{19}F NMR (377 MHz, acetonitrile-d$_3$, 25 °C) of the competition reaction between **1** and $[PPh_4][SbF_6]$ in acetonitrile. The signal for the $[SbF_6]^-$ ion is not present anymore and instead signals corresponding to the formation of $[Sb_2F_{11}]^-$ and other Sb−F species are visible. Furthermore, signals corresponding to the formation of a fluoride adduct of AFS, as well as HSO_3F and **2** can be found.

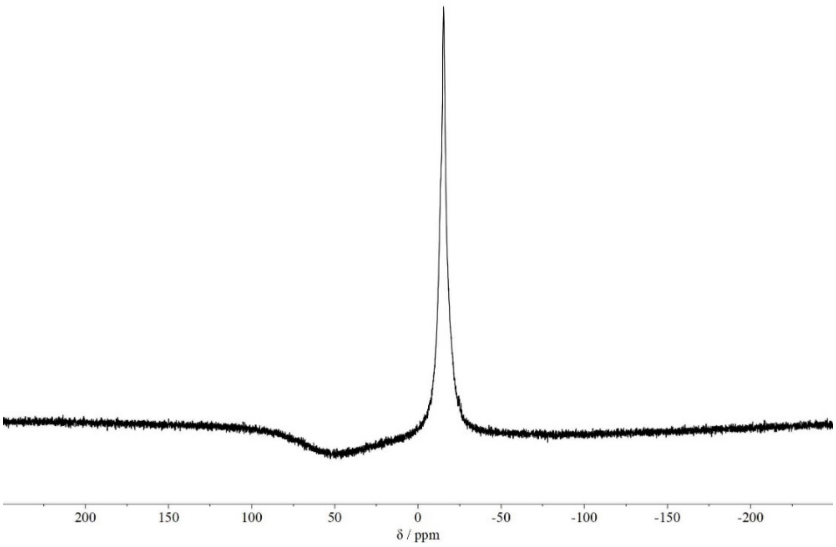

Figure S 15.: ^{27}Al NMR (107 MHz, acetonitrile-d_3, 25 °C) of the competition experiment between **1** and $[PPh_4][SbF_6]$ in acetonitrile.

4. Testing the reactivity of AFS

4.1. Halide Abstraction Reaction with Ph_3CCl.

A solution of Ph_3CCl (7 mg, 0.23 mmol, 0.92 eq) in DCM (1.0 mL) was added to **1** (88 mg, 0.25 mmol). Immediately, the solution turned bright yellow, and a bright yellow solid precipitated. The mixture was stirred for one week at room temperature. Afterward, all volatiles were pumped off and the remaining yellow solid was washed with *n*-pentane and dried in vacuo leading to the isolation of a bright yellow powder (127 mg, 92%).

^{1}H NMR (400 MHz, methylene chloride-d$_2$, 25 °C): δ = 8.34 (br, 3H, para-H, $[Ph_3C]^+$), 7.96 (br, 6H, meta-H, $[Ph_3C]^+$), 7.75 (br, 6H, ortho-H, $[Ph_3C]^+$) ppm. **^{19}F NMR (377 MHz, methylene chloride-d$_2$, 25 °C):** δ = 38.3 (s) ppm. **^{27}Al NMR (107 MHz, methylene chloride-d$_2$, 25 °C):** δ = −13 (s) ppm.

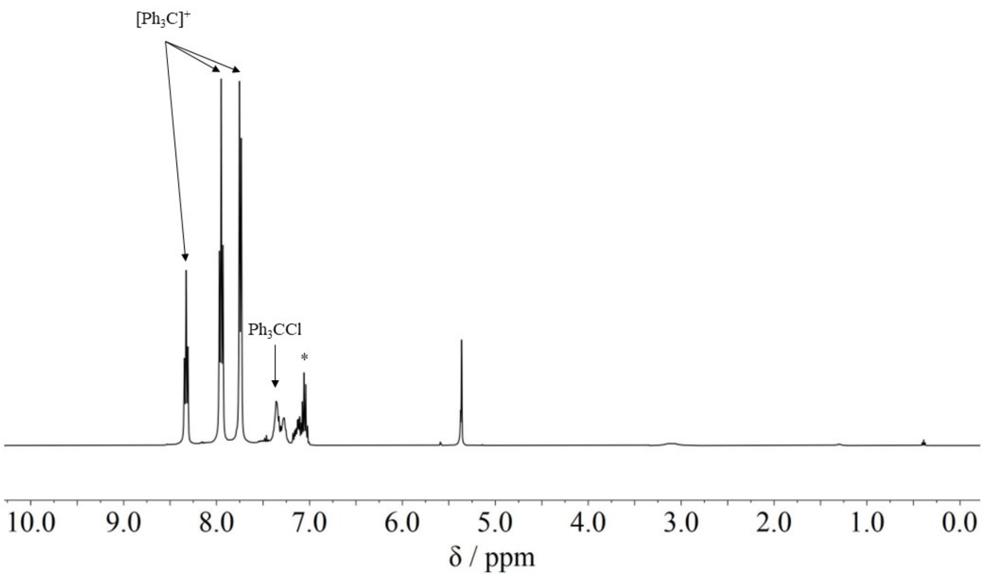

Figure S 16. ^{1}H NMR (400 MHz, methylene chloride-d$_2$, 25 °C) of chloride abstraction reaction between **1** and Ph_3CCl. The asterisk marks some unidentified side product.

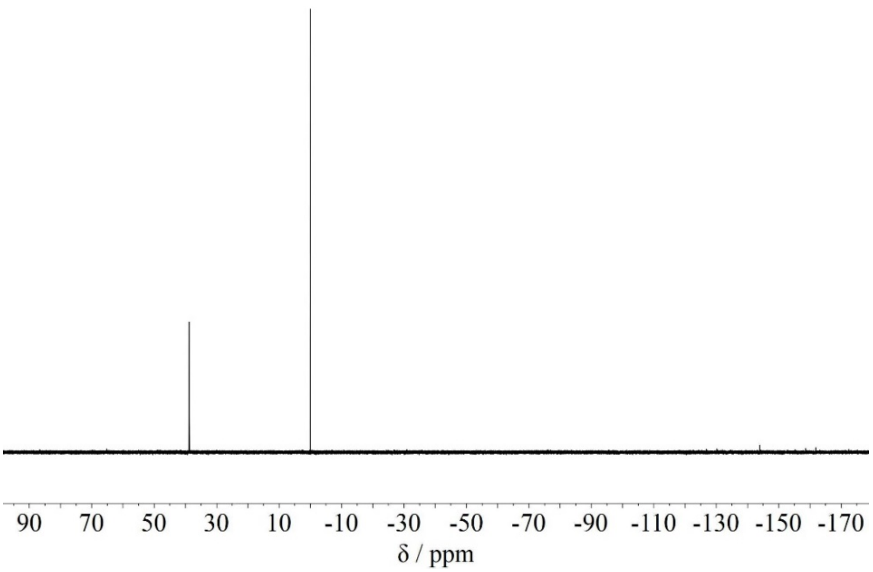

Figure S 17. ^{19}F NMR (377 MHz, methylene chloride-d_2, 25 °C) of chloride abstraction reaction between **1** and Ph_3CCl.

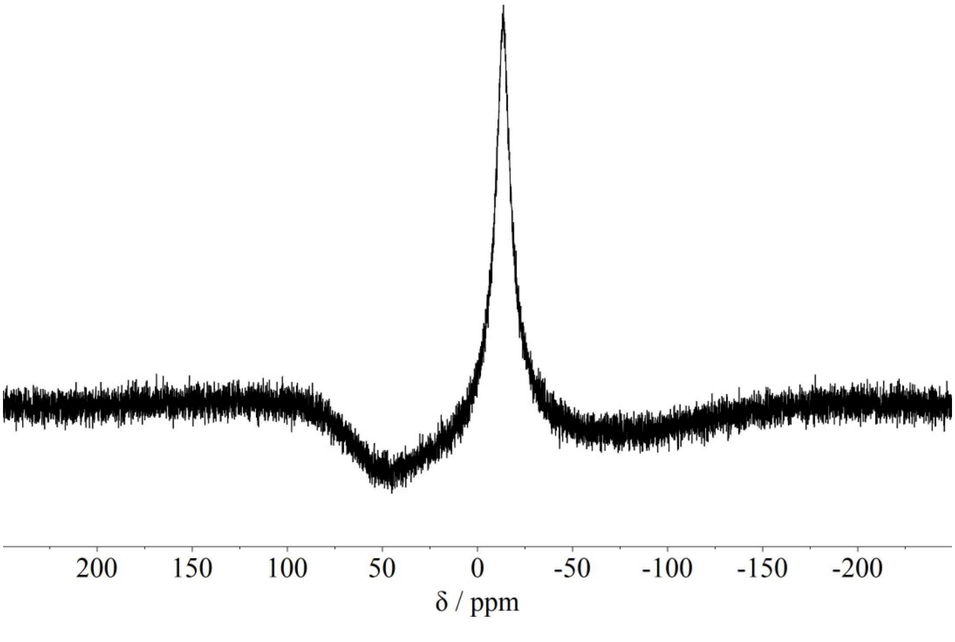

Figure S 18. ^{27}Al NMR (107 MHz, methylene chloride-d_2, 25 °C) of chloride abstraction reaction between **1** and Ph_3CCl.

4.2. Halide Abstraction Reaction with Ph^F_3CCl.

A suspension of **1** (12.5 mg, 0.038 mmol) in SO_2 (1 mL) was cooled to −80 °C. Afterwards, Ph^F_3CCl (21 mg, 0.038 mmol, 1.0 eq.) was added and the reaction mixture was slowly brought to room temperature, while vigorously stirred. During that time, the colorless suspension turned violet-red.

^{19}F NMR (377 MHz, SO_2, ext. acetone-d$_6$, 25 °C): δ = 43.9 (s), −138.6 (m), −149.4 (m), −160.1 (m).

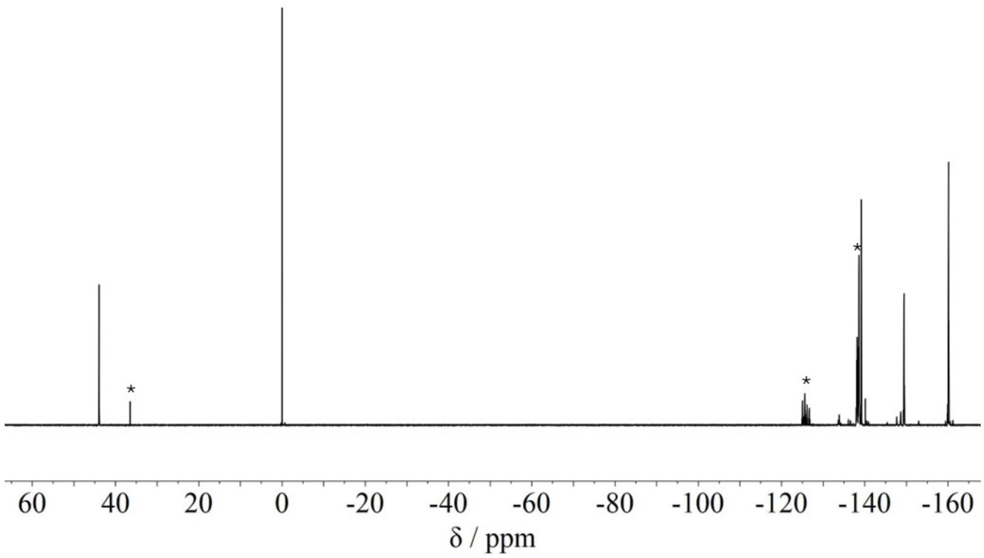

Figure S 19. ^{19}F NMR (377 MHz, SO_2, ext. acetone-d$_6$, 25 °C) of the chloride abstraction reaction between **1** and Ph^F_3CCl. Asterisks mark some unidentified side products.

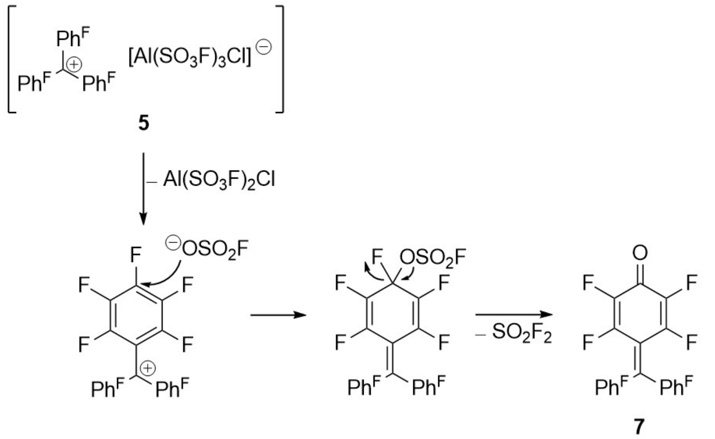

Figure S 20. Proposed mechanism for the formation of ketone **7**.

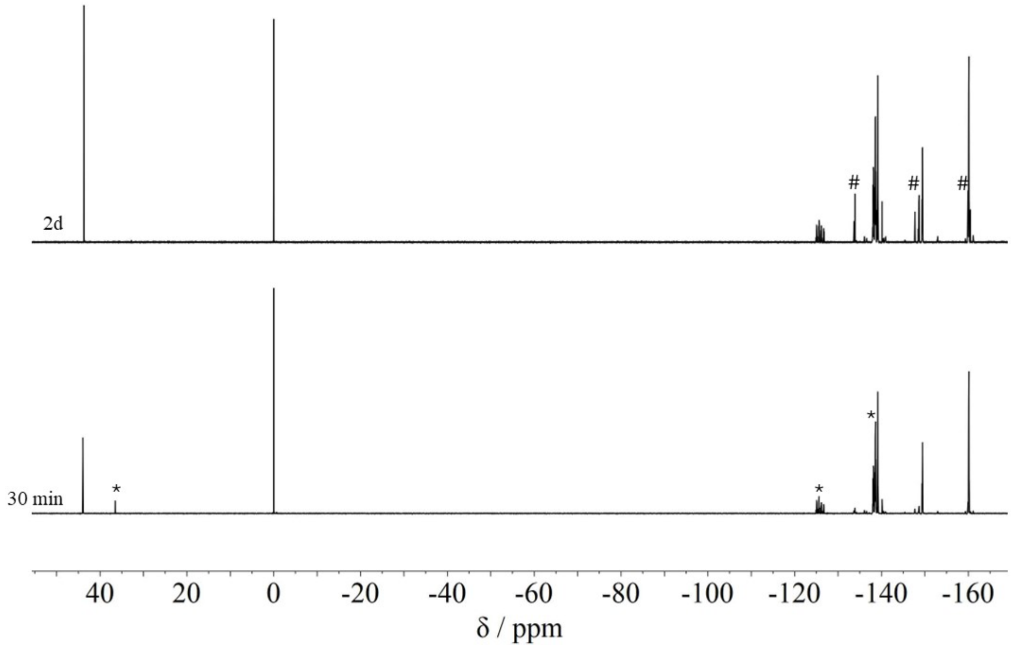

Figure S 21. ^{19}F NMR (377 MHz, SO_2, ext. acetone-d_6, 25 °C) of the reaction between **1** and $Ph^F{}_3CCl$. After some time, signals corresponding to ketone **7** appear (#) in addition to fluorosulfate **6**. Asterisks mark some unidentified side products.

Tab S1.: Calculated absorption maxima of **6** and **7** (B3LYP-D3(BJ)-def2-TZVP).

compound	Absorption maximum/ wavelength [nm]	fosc
**6**	252 ($S_0 \rightarrow S_4$)	0.091913997
**7**	426 ($S_0 \rightarrow S_2$)	0.482531664

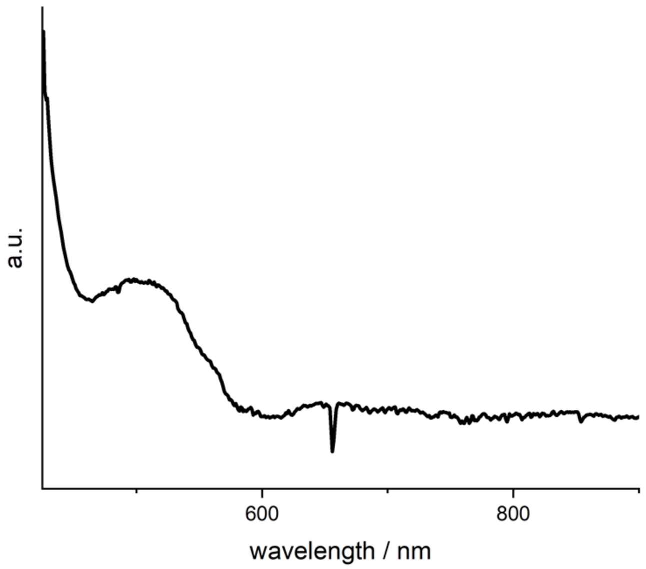

Figure S 22. UV-Vis spectrum of the reaction mixture of **1** and $Ph^{F}_{3}CCl$.

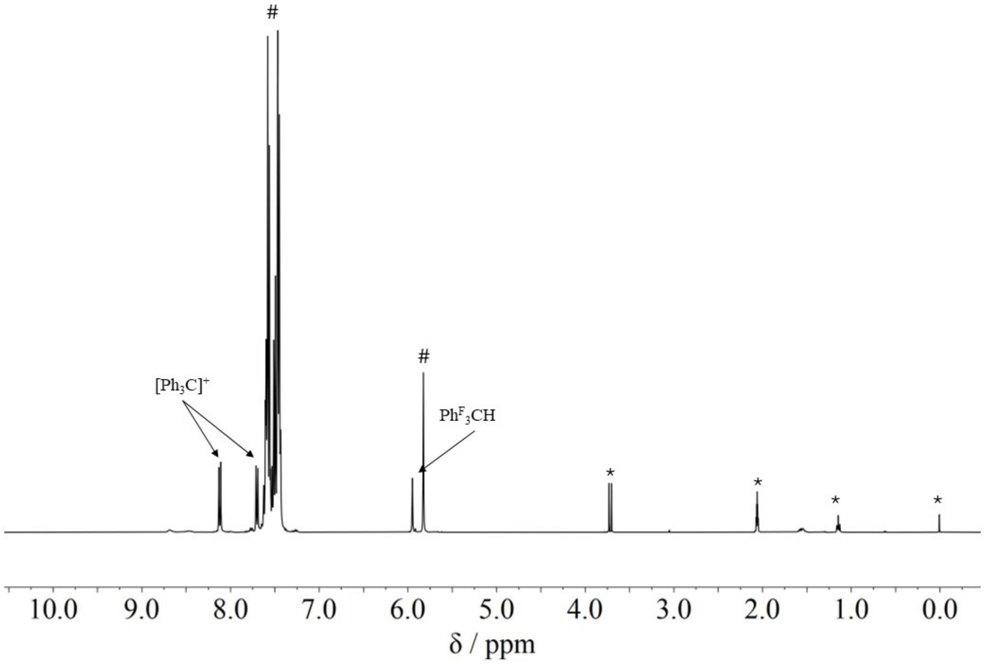

Figure S 23. ^{1}H NMR (400 MHz, SO_2, ext. acetone-d_6, 25 °C) after the addition of Ph_3CH to the reaction mixture of the reaction between **1** and $Ph^{F}_{3}CCl$. The signals of the formed trityl cation $[Ph_3C]^{+}$ are partly superimposed by the signals corresponding to unreacted Ph_3CH. The signal corresponding to perfluorotrityl methane is visible. Asterisks mark the signals of the capillary, and cross mark unreacted Ph_3CH.

4.3. Deoxygenation of Et_3PO

A solution of Et_3PO (6 mg, 0.44 mmol, 1.0 eq.) in CH_2Cl_2 was added at room temperature to a fresh sample of **1** (14 mg, 0.44 mmol), that was not dried overnight under high vacuum at 40 °C. A clear solution was obtained, that was stirred for one day. The reaction progress was monitored through ^{31}P and ^{19}F NMR spectroscopy. All volatiles were removed *in vacuo* leading to the isolation of a colorless, crystalline powder.

$^{31}P\ \{^1H\}$ NMR (162 MHz, methylene chloride-d_2, 25 °C): δ = 149.4 (d) ppm. **^{19}F NMR (377 MHz, methylene chloride-d_2, 25 °C):** δ = 38.5 (s) ppm. **^{27}Al NMR (107 MHz, methylene chloride-d_2, 25 °C):** δ = −12 (s) ppm.

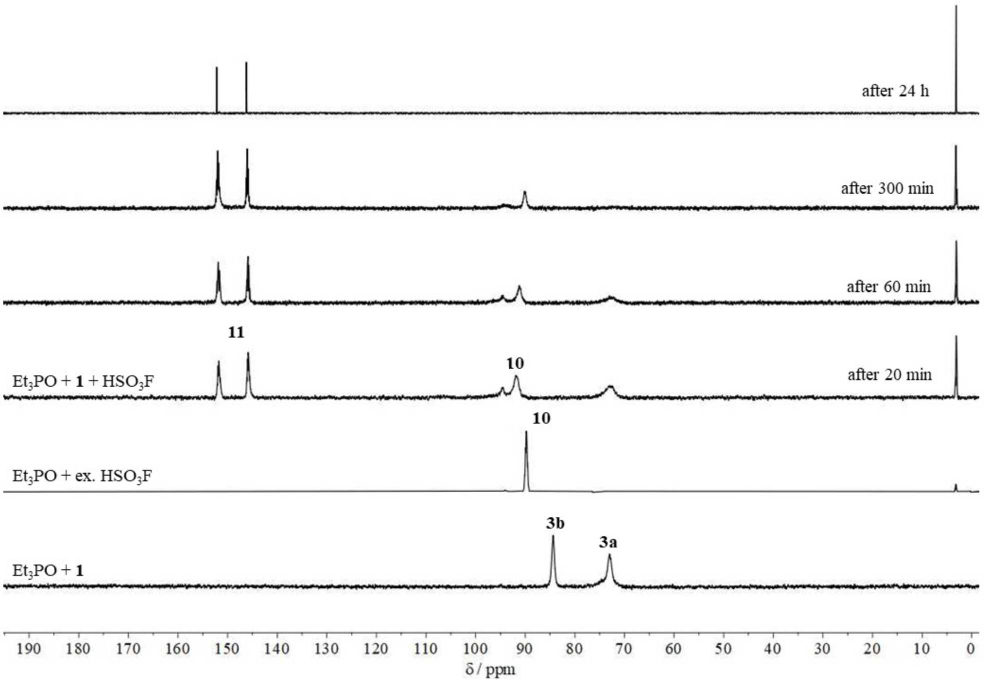

Figure S 24.: Stacked ^{31}P NMR (162 MHz, methylene chloride-d_2, 25 °C) spectra of the deoxygenation of Et_3PO by HSO_3F and **1**.

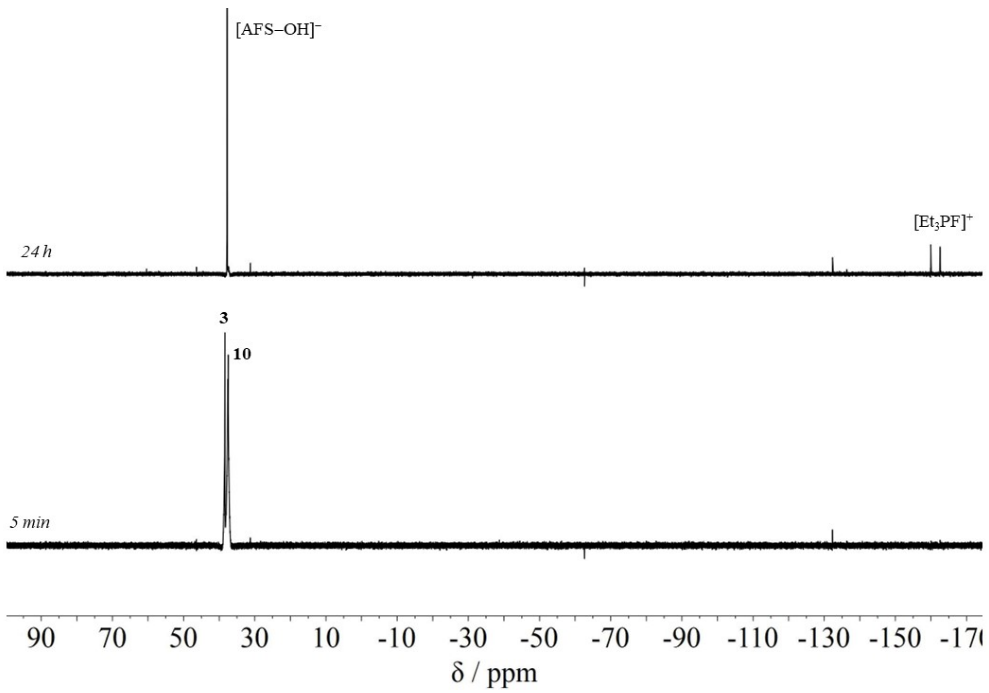

Figure S 25. ^{19}F NMR (377 MHz, methylene chloride-d_2, 25 °C) of the reaction between **1** and Et_3PO in the presence of residual HSO_3F. After 5 minutes signals are observed that can be attributed to the Gutmann-Beckett complex **3**, and phosphonium species **10**. After 24h only signals corresponding to the fluorophosphonium cation and its counter anion are visible.

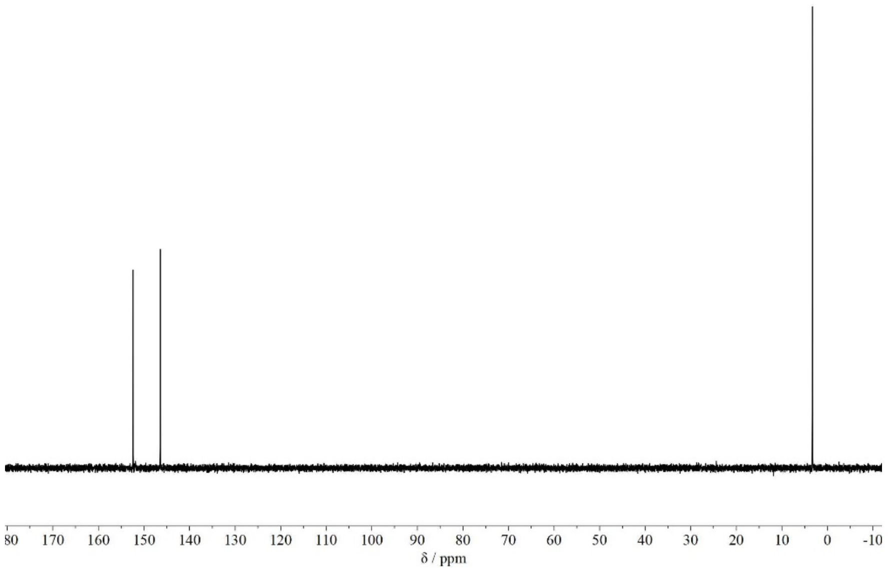

Figure S 26.: $^{31}P\{^1H\}$ NMR (162 MHz, methylene chloride-d_2, 25 °C) of fluorophosphonium species **11**.

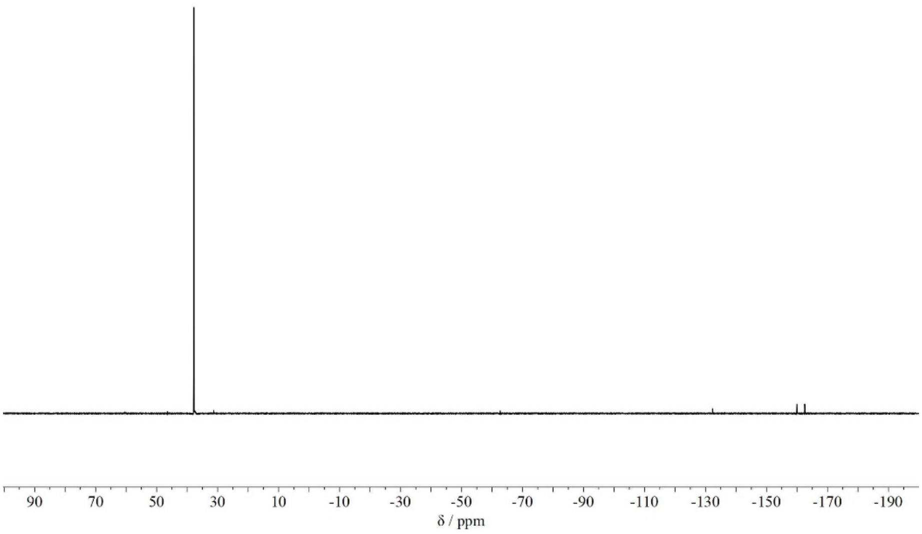

Figure S 27.: ^{19}F NMR (377 MHz, methylene chloride-d_2, 25 °C) of fluorophosphonium species **11**.

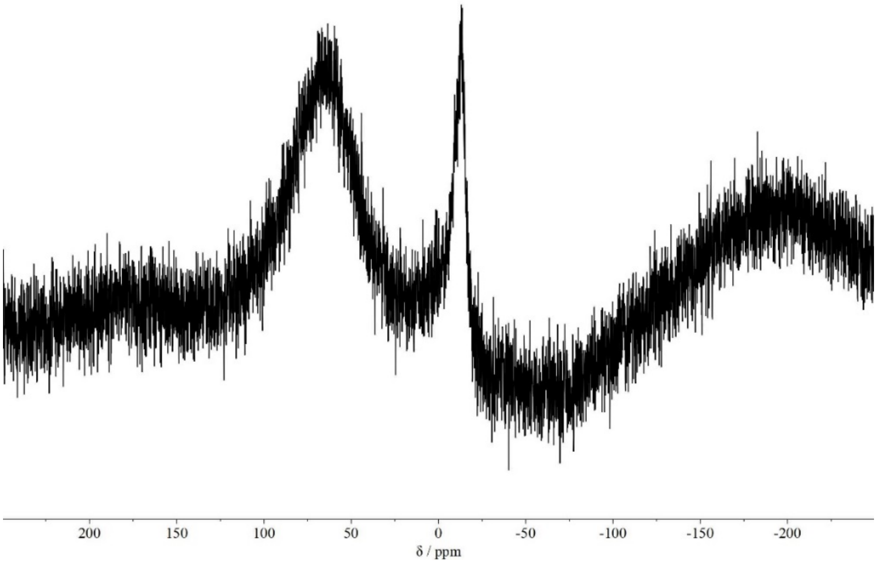

Figure S 28.: ^{27}Al NMR (107 MHz, methylene chloride-d_2, 25 °C) of fluorophosphonium species **11**.

5. Testing the catalytic activity

5.1. General procedure for C−F activation reactions

1 (30 mg or 10 mg) was placed in a *J. Young* NMR tube and combined with Et_3SiH (0.2 mmol). Then, the fluoroalkane (0.2 mmol) was added and a gas evolution was observed. DCM-d_2 was added to the reaction mixture. The NMR tube was vigorously shaken and the reaction progress was monitored via ^1H and ^{19}F NMR spectroscopy in different time intervals. A capillary, containing $CFCl_3$, was added as external standard.

Note: 30 mg of AFS (M = 324.17 g mol^{-1}) correspond to 46 mol% taking all deployed Al atoms into account; including bulk atoms (100% acidic sites). However, due to the heterogenous nature of the catalytic reaction only a fraction of the Al atoms will be catalytically active. For related catalysts (ACF, ACF teflate) around 10% acidic sites were assumed, following NH_3-TPD measurements which estimate the number of Lewis-acidic sites for HS-AlF_3 to be 0.978 mmol g^{-1} and for ACF roughly 1 mmol g^{-1}.[8]

Two comparative experiments using 10 mg of AFS (15 mol%, assuming 100% acidic sites) were performed both for 1-fluoropentane (0.2 mmol) and 1-fluoroheptane (0.2 mmol) in the presence of Et_3SiH (0.2 mmol) (Fig. S33). For 1-fluoropentane a conversion of 65% after 24 h was observed, for 1-fluoroheptane the yield was 49% after 24 h.

5.2. NMR spectra of C−F activation reactions

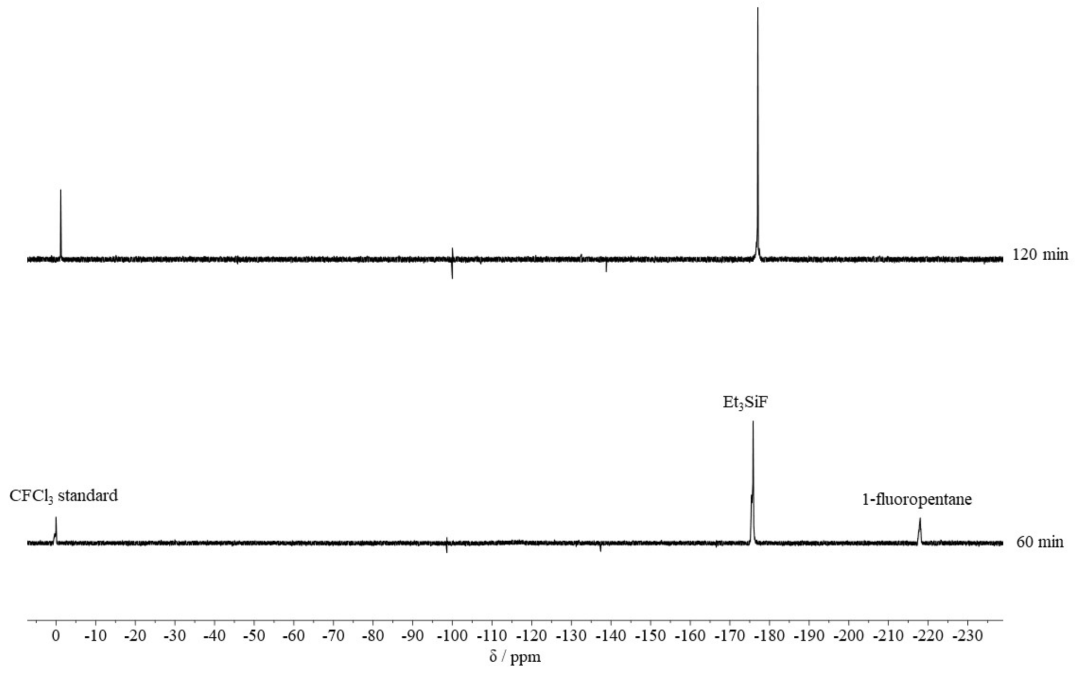

Figure S 29. Stacked ^{19}F NMR spectra (377 MHz, methylene chloride-d_2, 25 °C) of the reaction of 1-fluoro pentane with Et_3SiH in the presence of **1**. After 2 h 1-fluoropentane ($\delta(^{19}F) = -217$ ppm) is quantitatively consumed and Et_3SiF ($\delta(^{19}F) = -175$ ppm) is produced.

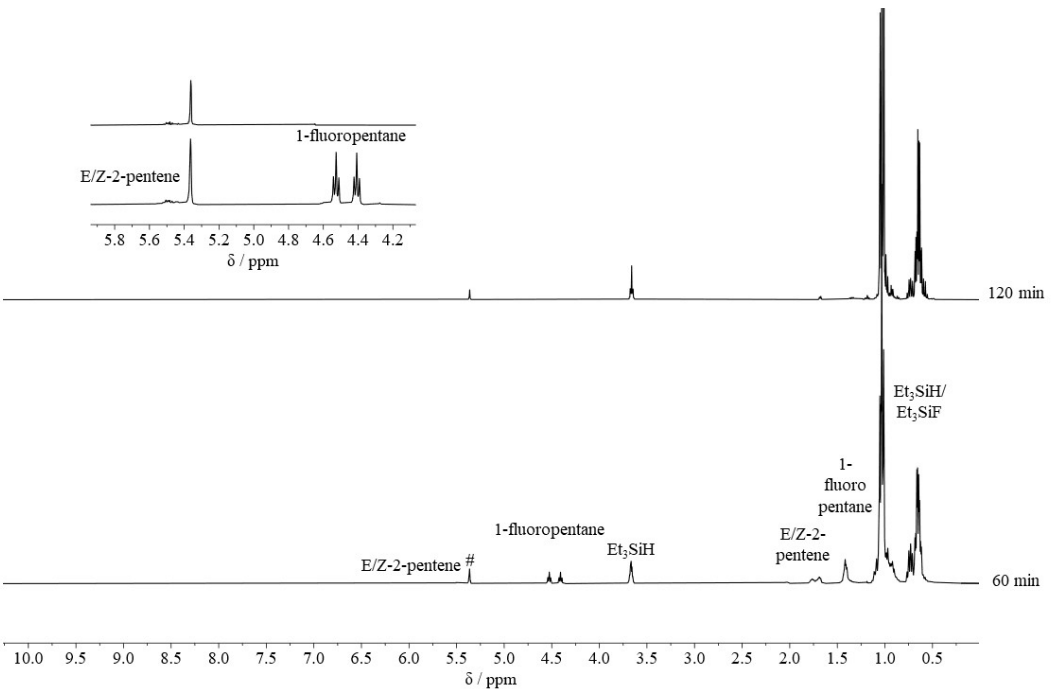

Figure S 30.: Stacked ^{1}H NMR spectra (400 MHz, methylene chloride-d$_2$, 25 °C) of the reaction of 1-fluoro pentane with Et_3SiH in the presence of **1**. After 2 h 1-fluoropentane is quantitatively consumed. The cross marks CD_2Cl_2.

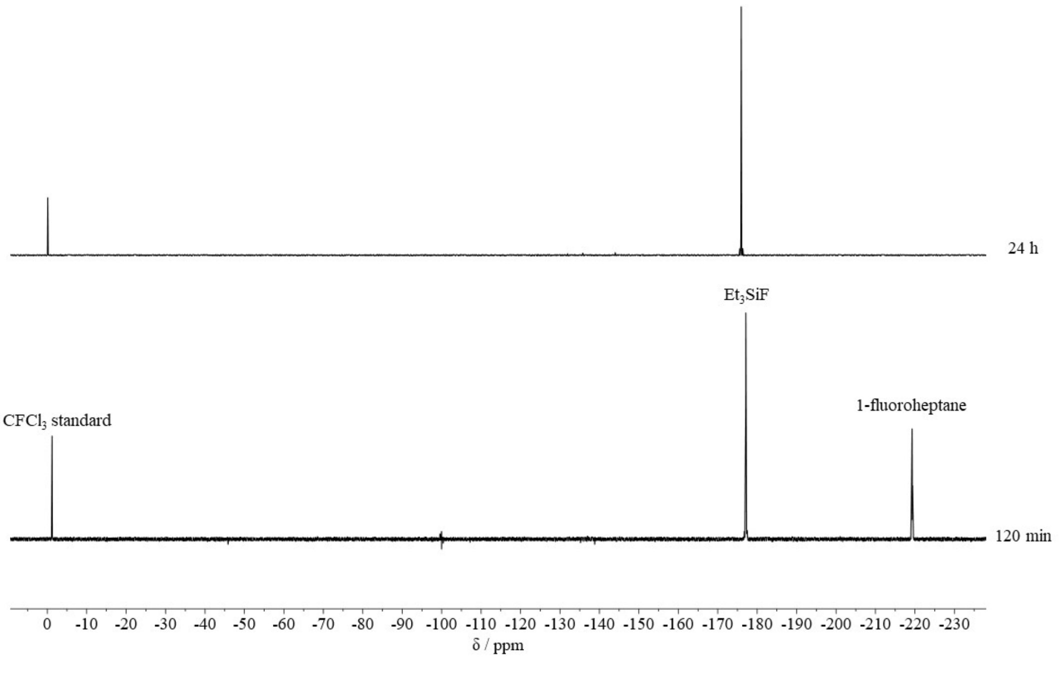

Figure S 31.: Stacked ^{19}F NMR spectra (377 MHz, methylene chloride-d$_2$, 25 °C) of the reaction of 1-fluoroheptane with Et_3SiH in the presence of **1**. After 24 h 1-fluoroheptane ($\delta(^{19}F) = -218$ ppm) is quantitatively consumed and Et_3SiF ($\delta(^{19}F) = -175$ ppm) is produced.

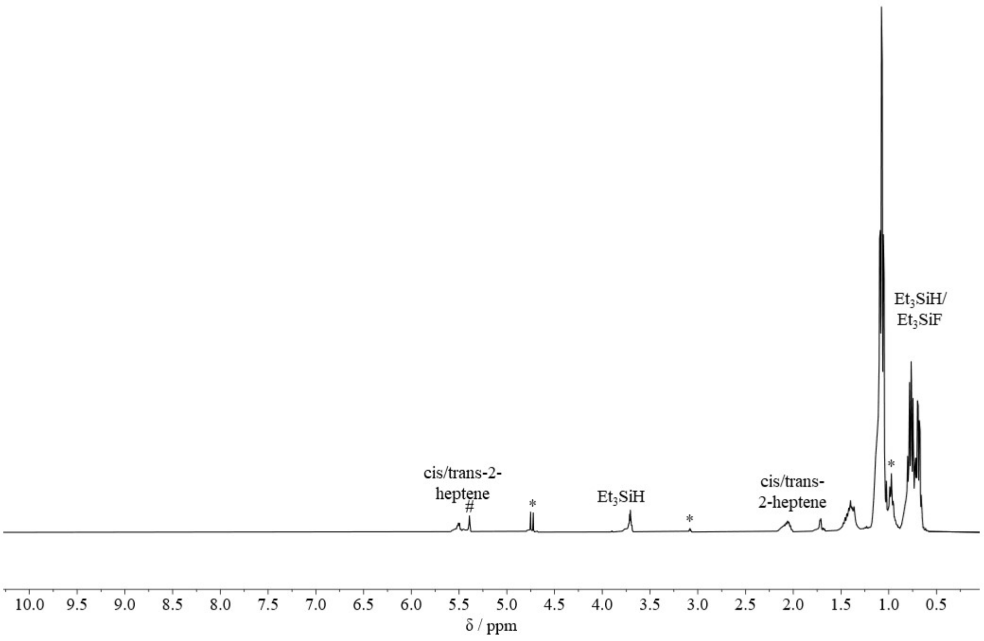

Figure S 32.: ^{1}H NMR spectrum (400 MHz, methylene chloride-d$_2$, 25 °C) of the reaction of 1-fluoroheptane with Et$_3$SiH in the presence of **1**. After 24 h 1-fluoroheptane is quantitatively consumed. Asterisks mark the external capillary. The cross marks CD_2Cl_2.

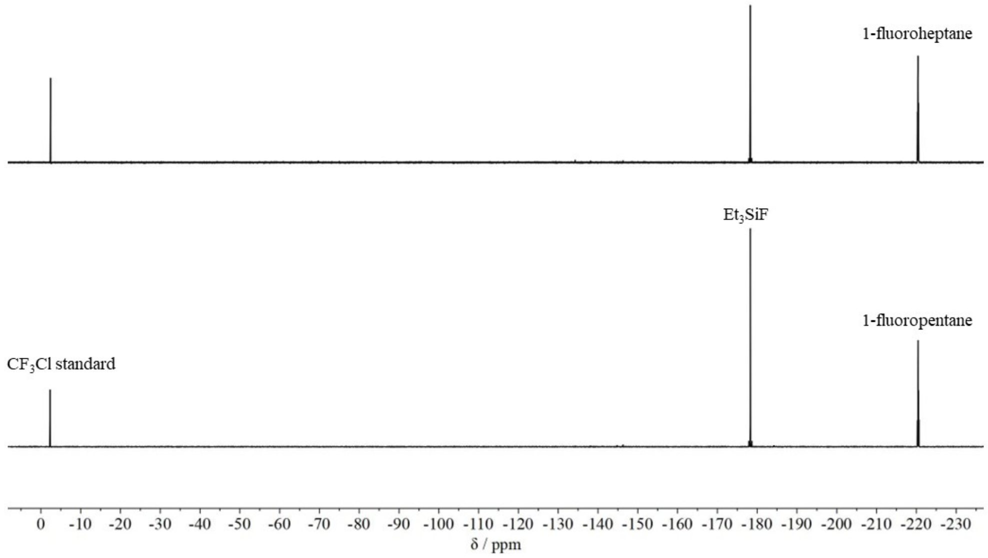

Figure S 33.: Stacked ^{19}F NMR spectra (377 MHz, methylene chloride-d$_2$, 25 °C) of the reaction of 1-fluoropentane/1-fluoroheptane with Et$_3$SiH in the presence of **1** (10 mg).

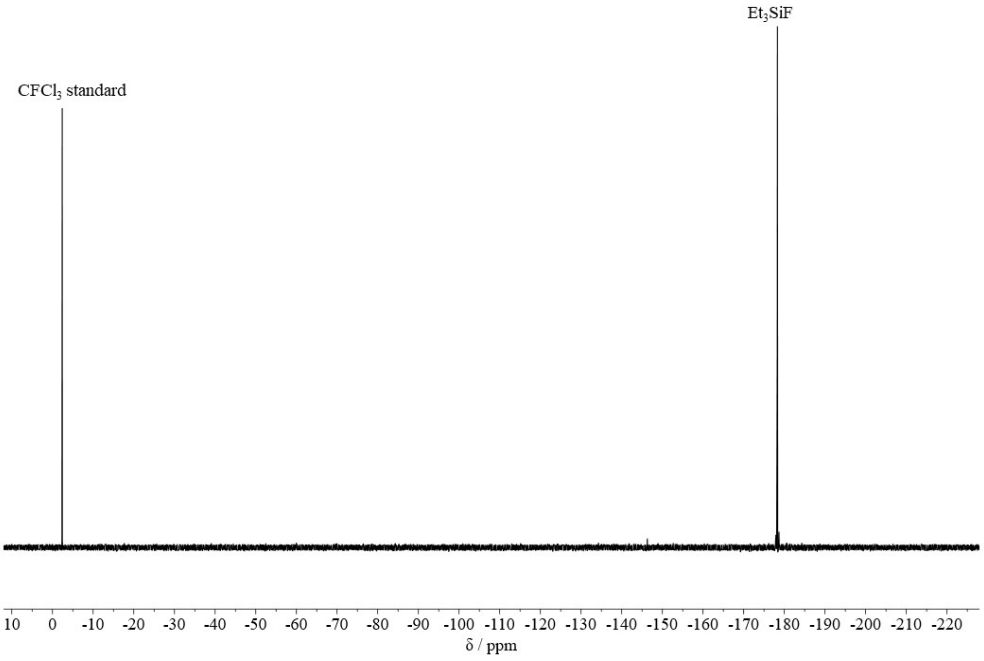

Figure S 34.: ^{19}F NMR spectrum (377 MHz, methylene chloride-d_2, 25 °C) of the reaction of 2-fluoropentane with Et_3SiH in the presence of **1**. After 1 h 2-fluoropentane ($\delta(^{19}F) = -173$ ppm) is quantitatively consumed and Et_3SiF ($\delta(^{19}F) = -175$ ppm) is produced.

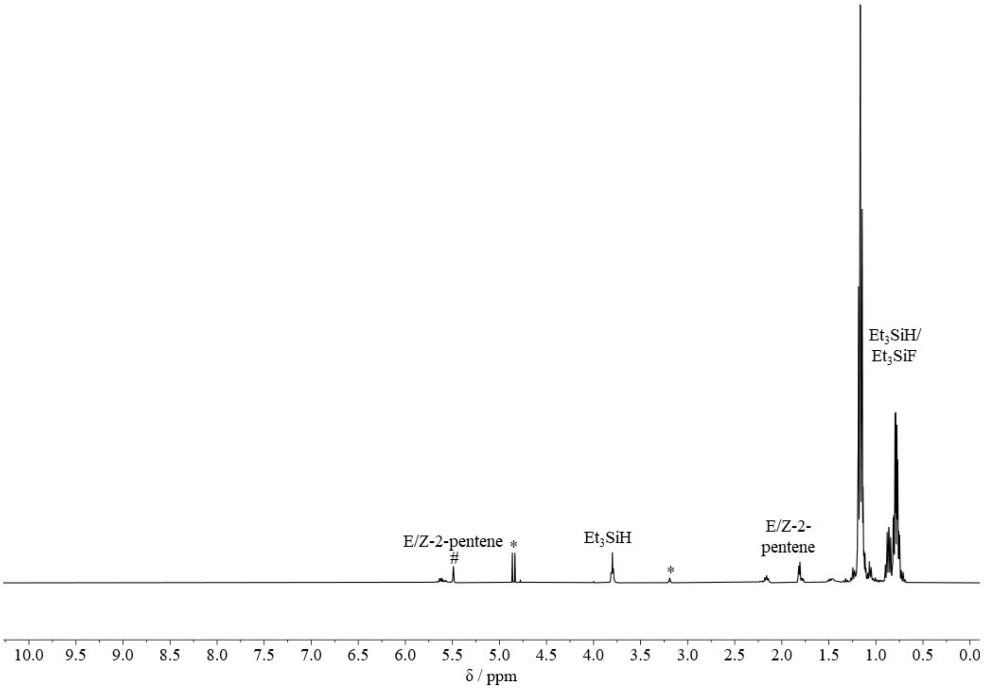

Figure S 35.: ^{1}H NMR spectrum (400 MHz, methylene chloride-d_2, 25 °C) of the reaction of 2-fluoropentane with Et_3SiH in the presence of **1**. After 1 h 2-fluoropentane is quantitatively consumed. Asterisk mark the external capillary. The cross marks CD_2Cl_2.

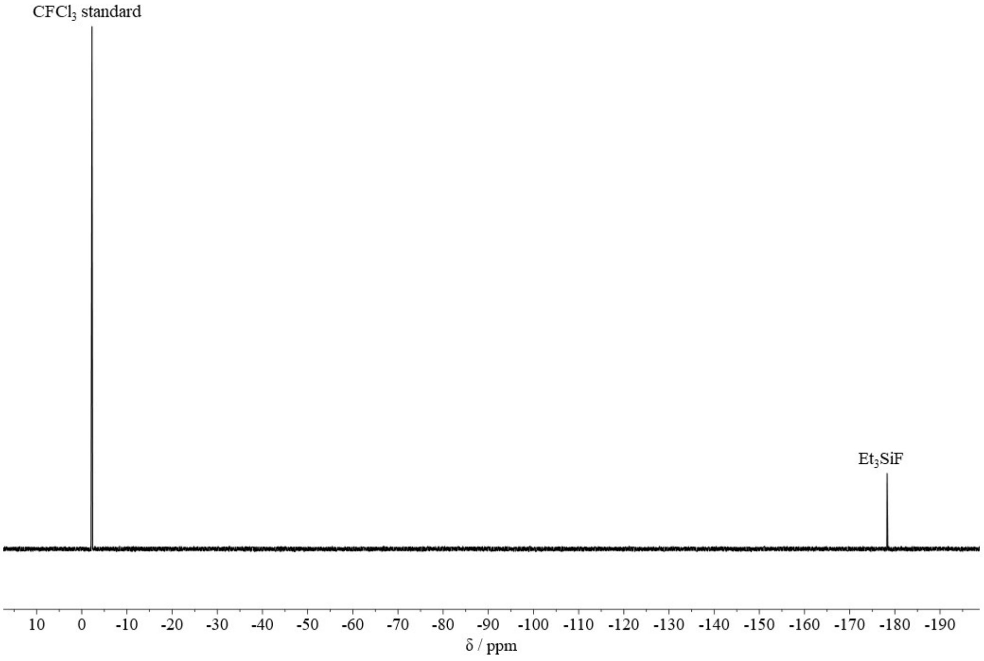

Figure S 36.: ^{19}F NMR spectrum (377 MHz, methylene chloride-d_2, 25 °C) of the reaction of 1-fluorocyclohexane with Et_3SiH in the presence of **1**. After 1 h 1-fluorocyclohexane ($\delta(^{19}F) = -172$ ppm) is quantitatively consumed and Et_3SiF ($\delta(^{19}F) = -175$ ppm) is produced.

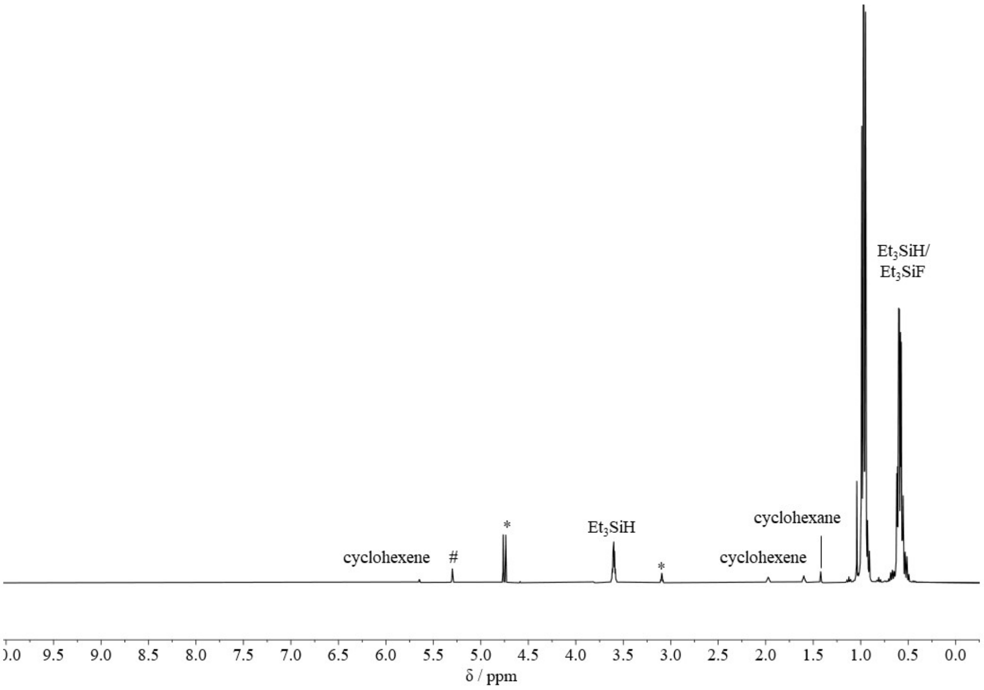

Figure S 37.: ^{1}H NMR spectrum (400 MHz, methylene chloride-d_2, 25 °C) of the reaction of 1-fluorocyclohexane with Et_3SiH in the presence of **1**. After 1 h 1-fluorocyclohexane $\delta(^{1}H) = 4.5$ ppm) is quantitatively consumed. Asterisk mark the external capillary. The cross marks CD_2Cl_2.

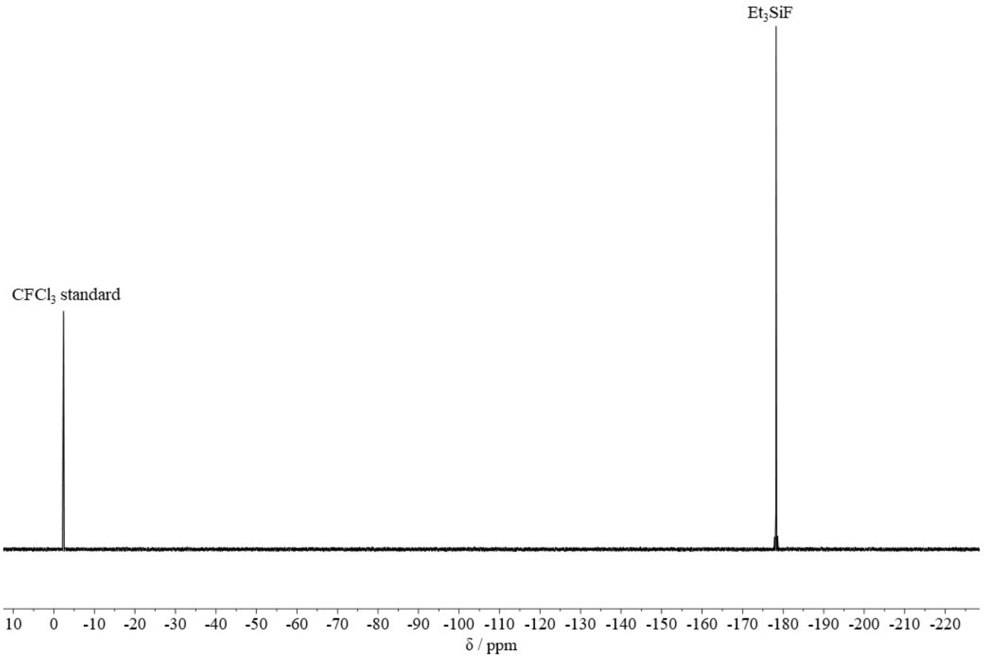

Figure S 38.: ^{19}F NMR spectrum (377 MHz, methylene chloride-d_2, 25 °C) of the reaction between Et_3SiH and 1-fluoroadamantane in the presence of **1**. After 1 h 1-fluoroadamantane ($\delta(^{19}F) = -128$ ppm) is completely consumed, and Et_3SiF ($\delta(^{19}F) = -175$ ppm) is produced.

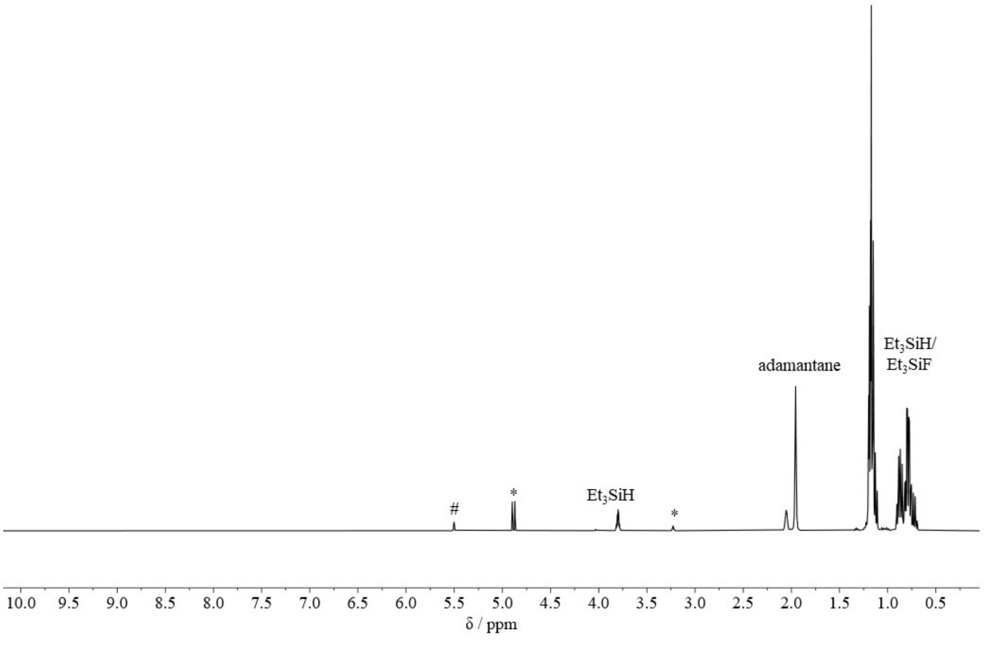

Figure S 39.: ^{1}H NMR spectrum (400 MHz, methylene chloride-d_2, 25 °C) of the reaction of 1-fluoroadamantane with Et_3SiH in the presence of **1**. After 1 h 1-fluoroadamantane is quantitatively consumed. Asterisk mark the external capillary. The cross marks CD_2Cl_2.

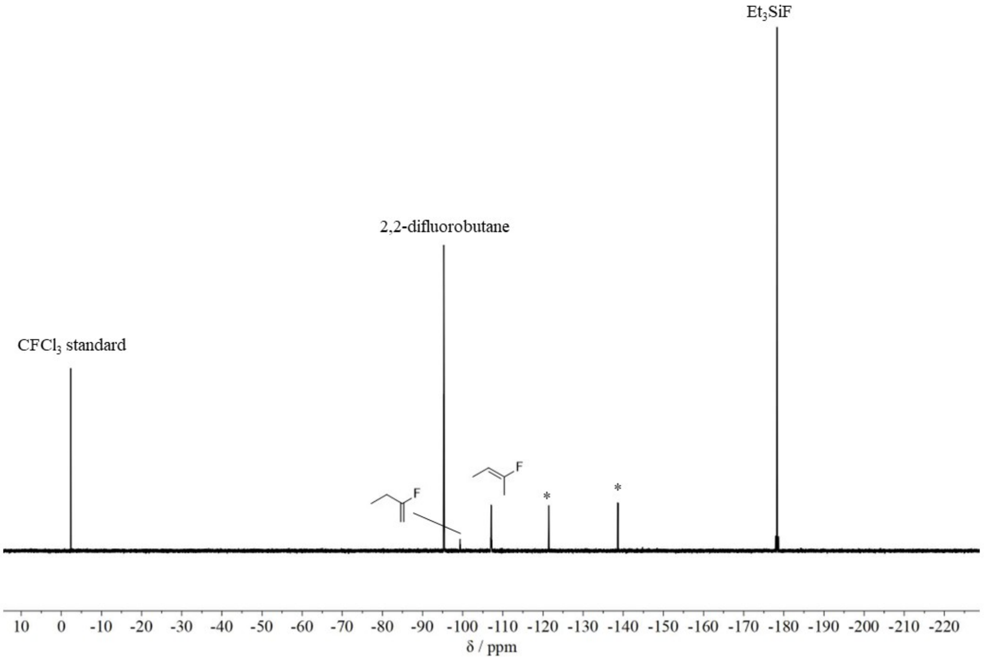

Figure S 40.: ^{19}F NMR spectrum (377 MHz, methylene chloride-d$_2$, 25 °C) of the reaction between Et_3SiH and 2,2-difluorobutane in the presence of **1**. After 24 h 2,2-difluorobutane (δ(^{19}F) = –93 ppm) is mostly consumed and Et_3SiF (δ(^{19}F) = –175 ppm) is produced. Asterisk mark some unidentified impurity in the 2,2-difluorobutane batch.

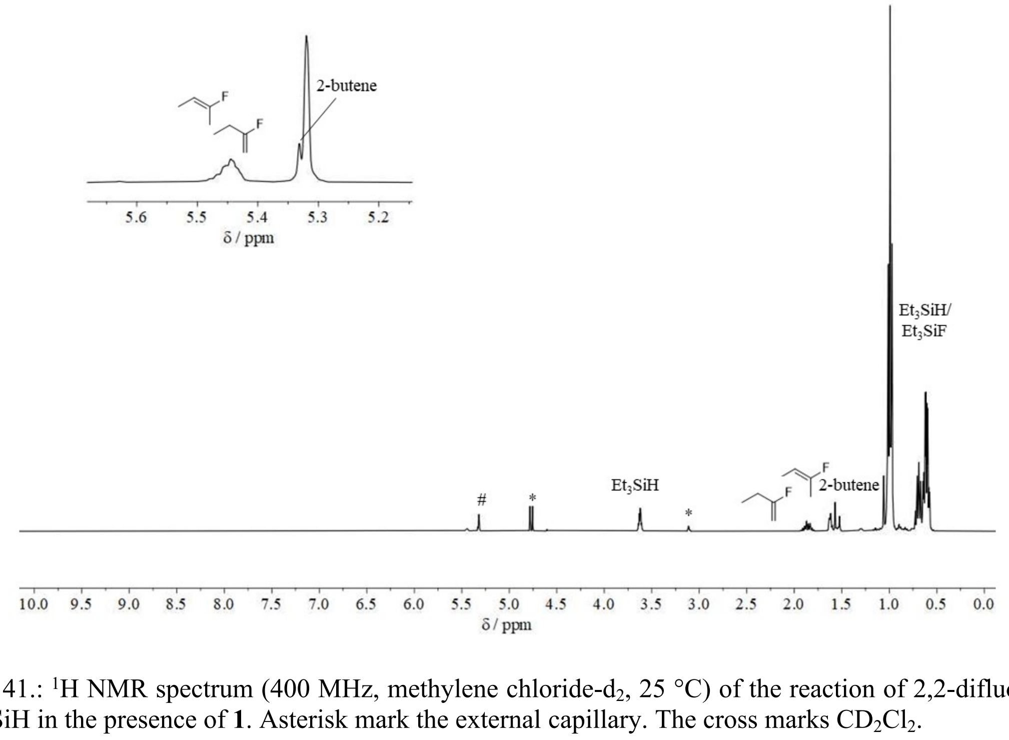

Figure S 41.: ^{1}H NMR spectrum (400 MHz, methylene chloride-d$_2$, 25 °C) of the reaction of 2,2-difluorobutane with Et_3SiH in the presence of **1**. Asterisk mark the external capillary. The cross marks CD_2Cl_2.

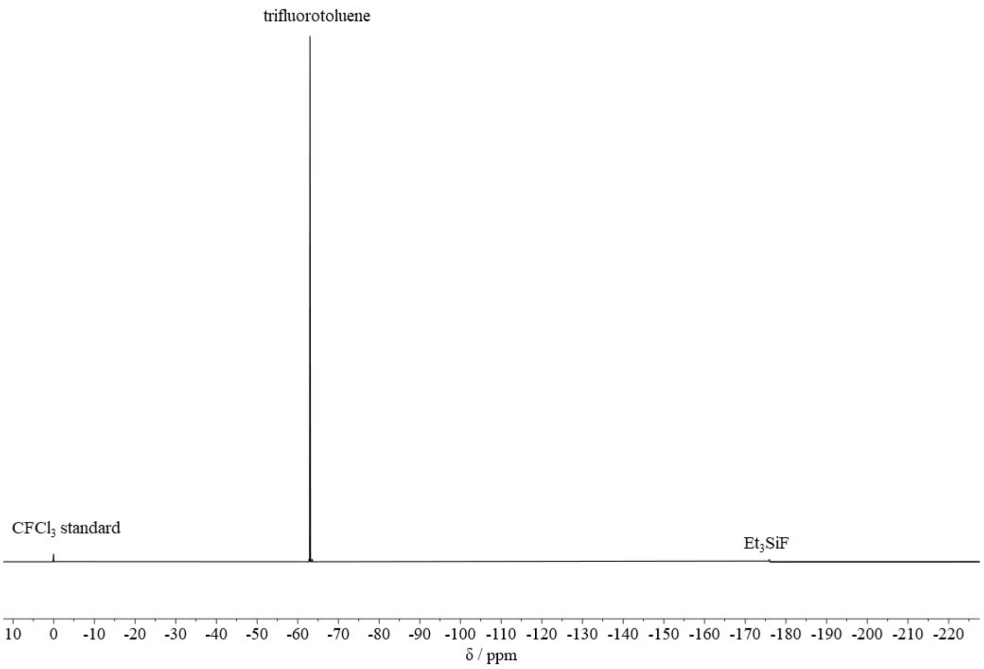

Figure S 42.: ^{19}F NMR spectrum (377 MHz, methylene chloride-d_2, 25 °C) of the reaction between Et_3SiH and trifluorotoluene in the presence of **1**. After 24 h trifluorotoluene (δ(^{19}F) = –62 ppm) is still present and only a small amount of Et_3SiF (δ(^{19}F) = –175 ppm) is produced.

6. Quantum-chemical calculations

Tab. S2: Optimized geometries of theoretically studied compounds. All xyz coordinates are given in angstroms and all SCF energies are given in Hartree at the B3LYP-D3(BJ)-TZVPP level of DFT. All structures represent minima on their respective potential energy surface.

**$Al(SO_3F)_3$**			
Energy = -2413.726072635			
Al	-0.0608026	0.0287625	-0.0251043
O	0.1172693	1.4618498	-1.3037380
O	0.3275075	-1.3641140	1.2553842
O	-1.5486551	-0.8243278	-0.9077580
O	-1.6589395	0.6450241	0.8589082
O	1.1150319	1.3411034	0.7658931
O	1.2946787	-1.0990682	-0.8157596
S	1.0420902	2.2371104	-0.4255962
S	1.3522895	-1.9971167	0.3745828
S	-2.5514475	-0.1746821	-0.0128949
O	-3.7007784	0.4143550	-0.5734028
O	0.8274142	3.6163994	-0.2419512
O	1.3366866	-3.3929125	0.1892044
F	-3.0334166	-1.3278619	0.9139715
F	2.4338468	2.0767150	-1.1045368
F	2.7072250	-1.6412362	1.0527977
**$[Al(SO_3F)_3]_2$**			
Energy = -4820.091498804			
F	-3.1646441	2.2474194	-1.4867399
S	-4.0944314	1.9008989	-0.2800075
F	-0.9109715	1.8958100	2.4112460
O	-4.3777565	3.0840339	0.4511811
O	-3.3278256	0.8762945	0.4672548
O	-0.6125469	0.7020312	0.3255862
F	-0.0074338	-1.1015598	-2.2568355
S	-0.3914250	0.5507961	1.7988263
O	-2.2933568	-0.7490120	-1.5496872
O	-5.2432861	1.2694122	-0.9821122
O	0.9161809	0.3302727	2.3020550
Al	-2.1354370	-0.5556413	0.3484874
S	-1.2500348	-1.8377555	-1.6539613
O	-7.0955635	2.4591479	2.3866214
O	-1.4812018	-0.4375191	2.1304063
O	-0.9222421	-2.0166853	-0.2054936
O	-7.7686248	1.5250911	0.1390598
O	-7.2802228	-0.0326368	-2.2263246
O	-1.5172508	-2.9667919	-2.4688848
O	-9.6844468	-0.7907292	-2.3295128
S	-7.3949408	1.3357587	1.5749330
Al	-6.5723696	0.0691806	-0.4642216
O	-3.4813270	-1.7487945	0.8385301
O	-6.3591512	0.2445070	1.4304591
S	-8.3623620	-1.0209255	-1.8703675
F	-8.6200430	0.6013321	2.2144011
O	-5.3837569	-1.3619406	-0.6323043
O	-8.0950768	-1.1878612	-0.4065092
S	-4.6037471	-2.3873361	0.1008332

F	-7.8676445	-2.3614629	-2.5128210
O	-4.2826073	-3.5438222	-0.6570631
F	-5.5432004	-2.7863967	1.2835647

$[Al(SO_3F)_3]_3$

Energy = -7230.163046261

F	-3.4874012	1.8716885	-1.6803038
S	-3.9522339	1.4807501	-0.2437490
F	-0.1781264	2.0894353	1.8784076
O	-4.5360239	2.6124430	0.3829482
O	-2.7158654	0.9658376	0.3822501
O	-0.0758996	0.4346246	0.1111807
F	-3.2115098	-1.6795587	-3.3462785
S	0.1613265	0.5900059	1.5878214
O	-2.1803542	-0.8975908	-1.3968493
O	-4.8579333	0.3317192	-0.5377146
O	1.4511138	0.3337174	2.1193047
Al	-1.6802497	-0.6093682	0.3691471
S	-1.8514935	-1.7479692	-2.5784655
O	-7.1491928	2.8694907	1.9113632
O	-1.0216529	-0.1687269	2.1270419
O	-0.8100854	-1.2802412	-3.4164080
O	-7.4487484	1.3907332	-0.1237672
O	-6.8927492	-0.9421999	-1.7549758
O	-1.7585956	-3.1738794	-2.1877365
O	-9.3599674	-1.3323552	-2.1311935
S	-7.4333678	1.5943510	1.3635405
Al	-6.5067752	-0.3162680	0.0031440
O	-3.1905405	-1.6065999	1.0611310
O	-6.5950435	0.4041771	1.7666613
S	-8.1971097	-1.5918788	-1.3637484
F	-8.8778698	1.2006037	1.8224381
O	-5.5979612	-1.8986794	0.6222479
O	-8.1874477	-1.3068910	0.1088122
S	-4.2652351	-2.4964703	0.6045991
F	-7.8962382	-3.1245538	-1.4639651
O	-3.9809618	-3.1988007	-0.6429661
F	-4.3467933	-3.5709717	1.7107567
Al	-2.5978091	-4.5781086	-1.2910040
O	-1.7917503	-4.2928738	0.3781977
O	-0.6120118	-2.1736099	0.3330162
S	-0.6012913	-3.5541346	0.8822964
F	0.5805892	-4.1741994	0.0711254
O	-0.3524051	-3.7042114	2.2692950
O	-3.7862183	-5.9443025	-0.5920448
O	-1.3236693	-5.7031640	-1.9373597
O	-3.9005881	-4.9649356	-2.6747622
O	-0.6599109	-7.0733557	-0.0066135
S	-4.5963573	-6.0156874	-1.8575652
S	-0.6913519	-6.9930756	-1.4402512
O	-4.8874945	-7.2770433	-2.4361590
O	0.4661190	-7.3136896	-2.2202190
F	-5.9646964	-5.3581324	-1.4655653
F	-1.8177190	-8.0275908	-1.8976714

$[Al(SO_3F)_3F]^-$

Energy = -2513.783452916

Al	0.2887438	0.1456634	0.7230048
O	-0.1298317	3.3033522	0.0899181
O	0.6509494	-1.5170181	0.2128051
O	-2.3080961	-0.2914719	-1.3464494
O	-1.4759118	0.2026733	0.8941075
O	0.8192301	1.1736461	-0.6201299
O	3.0908456	-1.5950756	0.1973620
S	0.5977682	2.6368808	-0.9412267
S	1.8969174	-2.3743227	0.2160562
S	-2.6518945	-0.2054574	0.0343039
O	-3.8395529	0.4543654	0.4557098
O	1.7650444	3.2207932	-1.5073631
O	1.7410955	-3.4959004	-0.6449630
F	-2.8143380	-1.7094947	0.5233578
F	-0.4181696	2.4798899	-2.1444336
F	1.8006735	-2.9621865	1.6905350
F	0.9865266	0.5336629	2.1674054

$Ph^F{}_3COSO_2F$ (6)

Energy = -2942.038312369

C	-0.2937518	-0.0846147	-0.3630600
C	0.1645048	1.3803427	-0.2451208
C	-1.0648593	-0.6408265	0.8356856
C	0.9764791	-0.8709159	-0.7424317
C	1.6844017	-1.7132899	0.1271987
C	0.4507123	3.6765792	-1.0244978
C	1.5879447	2.9648415	0.9720486
F	1.2685573	-1.9591653	1.3687777
F	-0.8683340	2.2149570	-2.2423909
F	-2.4511042	-3.8869485	1.7925572
C	2.8643122	-2.3495587	-0.2624375
F	2.3928987	3.2192393	1.9971421
F	1.3372272	0.7663286	1.7136489
C	1.5488824	-0.6514085	-2.0080866
F	3.2183409	-1.0553992	-3.6246561
F	4.5081186	-2.7386254	-1.9067350
C	2.7217073	-1.2846722	-2.4140303
C	1.2988305	3.9633853	0.0406698
F	1.8196925	5.1740341	0.1757728
C	3.3875231	-2.1378112	-1.5354224
F	0.1697571	4.6166958	-1.9202136
F	3.4935084	-3.1527963	0.5887958
C	-1.6084239	0.1339150	1.8693955
C	-1.4029366	-2.0050126	0.8315870
C	-2.1708321	-2.5893185	1.8319514
F	-3.4144924	-2.3343393	3.8183939
F	0.9982712	0.2034758	-2.8680492
F	-2.8613134	0.3326044	3.8575168
C	-2.3780302	-0.4349924	2.8870457
C	1.0350267	1.7006048	0.8076715
F	-0.9770748	-2.7938881	-0.1537378
C	-2.6720329	-1.7955935	2.8646992
C	-0.0931409	2.3966689	-1.1722489
F	-1.4269826	1.4494195	1.9406961
O	-1.1605806	-0.2836362	-1.5431235
F	-3.3845878	-1.1472910	-1.0733499
S	-2.6901130	0.2024878	-1.6066438

O	-2.9763791	1.2133662	-0.6167805
O	-3.0317272	0.3011582	-2.9982380

$C_{19}H_{10}F_6O$ (7)

Energy = -2194.358624205

C	0.1149056	-0.0919475	-0.0594644
C	0.0812146	1.3731531	-0.2527282
C	-1.0283690	-0.6280028	0.7089551
C	1.1288421	-0.8907942	-0.5530378
C	1.3738788	-2.2458730	-0.0681878
C	-1.1724778	3.4070359	-0.7700104
C	1.1135150	3.5719876	0.0067984
F	0.6401789	-2.7107050	0.9410333
F	-2.1628618	1.3047957	-1.0166752
F	-3.6722931	-3.1305690	0.4044597
C	2.3707808	-3.0299946	-0.5441098
F	2.1608731	4.3164544	0.3443874
F	2.3098482	1.6284032	0.5086162
C	2.0436048	-0.4481452	-1.6013305
F	3.8109467	-0.7996417	-3.0799702
O	4.1889697	-3.2964215	-2.0371445
C	3.0353970	-1.2295531	-2.0921731
C	-0.0667850	4.1851543	-0.4223140
F	-0.1329993	5.5048771	-0.5062513
C	3.2899410	-2.5904810	-1.6020217
F	-2.2890178	3.9905415	-1.1913124
F	2.5838883	-4.2379536	-0.0366670
C	-1.4460098	-0.0171630	1.9027149
C	-1.8085394	-1.6903949	0.2233783
C	-2.9459864	-2.1306200	0.8920669
F	-4.4068339	-1.9261541	2.7298455
F	1.8521752	0.7448248	-2.1609350
F	-2.9315474	0.1294082	3.7344358
C	-2.5738088	-0.4521739	2.5949796
C	1.1770518	2.1846667	0.0843940
F	-1.4739550	-2.2938039	-0.9152785
C	-3.3297939	-1.5082016	2.0830351
C	-1.0931167	2.0197764	-0.6712414
F	-0.7416165	0.9875148	2.4217530

7. Literature

[1] K. F. Hoffmann, D. Battke, P. Golz, S. M. Rupf, M. Malischewski, S. Riedel, *Angew. Chem. Int. Ed.* **2022**, *61*, e202203777.

[2] R. Ahlrichs, M. Bär, M. Häser, H. Horn, C. Kölmel, *Chem. Phys. Lett.* **1989**, *162*, 165.

[3] a) R. A. F. Weigend, *Phys. Chem. Chem. Phys.* **2005**, *7*, 3297; b) A. Schäfer, H. Horn, R. Ahlrichs, *J. Chem. Phys.* **1992**, *97*, 2571; c) A. D. Becke, *Phys. Rev. A* **1988**, *38*, 3098; d) S. H. Vosko, L. Wilk, M. Nusair, *Can. J. Phys.* **1980**, *58*, 1200; e) C. Lee, W. Yang, R. G. Parr, *Phys. Rev. B* **1988**, *37*, 785; f) J. P. Perdew, *Phys. Rev. B* **1986**, *33*, 8822.

[4] F. Neese, *Wiley Interdiscip. Rev. Comp. Mol. Sci.* **2018**, *8*, e1327.

[5] a) G. S. A. Klamt, *J. Chem. Soc. Perkin Trans.* **1993**, *2*, 799; b) M. C. V. Barone, *J. Phys. Chem. A* **1998**, *102*, 1995.

[6] S. Singh, R. D. Verma, *Polyhedron* **1983**, *2*, 1209.

[7] K. F. Hoffmann, A. Wiesner, S. S. Steinhauer, S. Riedel, *Chem. Eur. J.* **2022**, *28*, e202201958.

[8] a) J. K. Murthy, U. Gross, S. Rüdiger, V. V. Rao, V. V. Kumar, A. Wander, C. L. Bailey, M. N. Harrison, E. Kemnitz, *J. Phys. Chem. B* **2006**, *110*, 8314; b) S. M. Coman, P. Patil, S. Wuttke, E. Kemnitz, *Chem. Commun.* **2009**, *4*, 460; c) M. H. G. Prechtl, M. Teltewskoi, A. Dimitrov, E. Kemnitz, T. Braun, *Chem. Eur. J.* **2011**, *17*, 14385.
